# Effect of Exercise Training on Body Composition and Inflammatory Cytokine Levels in Overweight and Obese Individuals: A Systematic Review and Network Meta-Analysis

**DOI:** 10.3389/fimmu.2022.921085

**Published:** 2022-06-23

**Authors:** Shengya Wang, Huayi Zhou, Changtao Zhao, Hui He

**Affiliations:** ^1^ Department of Exercise Physiology, Beijing Sport University, Beijing, China; ^2^ Department of Physical Health and Arts Education, Ministry of Education, Beijing, China; ^3^ China Institute of Sport and Health Science, Beijing Sport University, Beijing, China

**Keywords:** exercise, body composition, inflammatory cytokine, overweight and obese individuals, network meta-analysis

## Abstract

**Objective:**

This study aimed to compare and rank the effectiveness of aerobic exercise (AE), resistance training (RT), combined aerobic and resistance training (CT), and high-intensity interval training (HIIT) on body composition and inflammatory cytokine levels in overweight and obese individuals by using network meta-analysis (NMA).

**Methods:**

We searched the PubMed, Cochrane, Embase, Web of Science, and EBSCO databases to identify randomized controlled trials investigating the effects of exercise training on inflammatory cytokines in overweight and obese patients. The retrieval period was from inception to November 2021. Two reviewers independently screened the retrieved articles, extracted the pertinent data, and assessed the risk of bias of the included studies; then, they used Stata 16.0 and Review Manager 5.3 to perform an NMA.

**Results:**

A total of 38 studies involving 1317 patients were included in this study. The results of the NMA indicated that AE had the greatest effect on weight loss (SUCRA=78.3; SMD=−0.51, 95% CI: −0.70, −0.33); CT had the greatest effect on reducing body mass index (SUCRA=70.7; SMD=−0.46, 95% CI: −0.81, −0.10), waist circumference (SUCRA=93.4; SMD=−1.86, 95% CI: −2.80, −0.93), percentage body fat (SUCRA=79.6; SMD=−1.38, 95% CI: −2.29, −0.48), interleukin-6 level (SUCRA=86.4; SMD=−1.98, 95% CI: −3.87, −0.09), and tumor necrosis factor-α level (SUCRA=79.4; SMD=−2.08, 95% CI: −3.75, −0.42); AE (SMD=0.51, 95% CI: −1.68, 2.69), RT (SMD=0.15, 95% CI: −3.01, 3.32), CT (SMD=1.78, 95% CI: −1.35, 4.92), and HIIT (SMD=2.29, 95% CI: −1.27, 5.86) did not significantly increase the adiponectin level.

**Conclusion:**

The current results suggest that CT is the best exercise modality for improving body composition and inflammatory status in overweight and obese individuals. More rigorous randomized control trials are needed for further validation.

**Systematic Review Registration:**

https://www.crd.york.ac.uk/prospero/, identifier CRD42022303165.

## 1 Introduction

Obesity prevalence worldwide has risen to the pandemic levels over the past 50 years (1). Approximately more than 2.1 billion adults are overweight or obese, of whom 1.5 billion are overweight and 640 million are obese (2). Based on the current trends, the global obesity rate will reach 18% in men and will exceed 21% in women by 2025 (3). Obesity has become one of the major health problems threatening the world today, and it is closely related to a range of diseases, such as cardiometabolic, digestive, respiratory, musculoskeletal, neurological, and infectious diseases (4, 5). The global economic cost of obesity and its complications is estimated to be US$2 trillion (2).

The adipose tissue is an organ specialized for long-term energy storage, and it grows through the increase in the number of adipocytes and in the size of each adipocyte when there is a surplus of nutrients (6). The adipose tissue plays an important role in systemic metabolic integration given its ability to produce and release a variety of inflammatory cytokines, such as leptin, adiponectin, tumor necrosis factor-α (TNF-α), and interleukin-6 (IL-6) (7, 8). When there is excessive adipose tissue mass, the unbalanced expression of pro- and anti-inflammatory adipokines may result in a metabolic dysfunction (7). On the one hand, the adipose tissue of obese individuals is infiltrated by a large number of macrophages (7). On the other hand, free fatty acid exposure promotes the change in macrophage phenotype from the anti-inflammatory M2 type to the pro-inflammatory M1 type; M1 macrophages in turn promote the production of pro-inflammatory cytokines (9, 10). These cytokines could amplify inflammation locally and distally by stimulating the secretion of pro-inflammatory molecules from other tissues, leading to systemic low-grade chronic inflammation (11). Thus, obesity is often accompanied by chronic low-grade inflammation. It is worth noting that obesity-induced inflammation involves multiple organs, including adipose, heart, skeletal muscle, pancreas, liver, and brain (12). Moreover, it can lead to several diseases, such as cardiovascular disease, diabetes mellitus, nephropathy, nonalcoholic fatty liver disease, cancer, autoimmune, and neurodegenerative disorders, which severely burden global health (13).

Obesity interventions mainly include lifestyle changes, dietary restrictions, increased physical activity, use of drugs, and surgery, when necessary (14). Among the lifestyle interventions, increased physical activity is important for obesity management (14). The beneficial effects of exercise training on body composition have been studied in the existing network meta-analysis. They found aerobic exercise as well as combined aerobic and resistance training are better forms of exercise for improving anthropometric outcomes (15, 16). More importantly, regular exercise training plays an essential role in reducing the risk of chronic metabolic and cardiorespiratory diseases partly due to the anti-inflammatory effects of exercise (17). Many meta-analyses and systematic reviews have studied the effect of exercise training on inflammatory cytokines, focusing on people with type 2 diabetes mellitus, metabolic syndrome, middle-aged and older adults, cancer survivors, and others (18–21). They found that exercise training can improve the level of related inflammatory markers in these groups. Obesity is closely related to the above diseases. Therefore, it is of great significance to study the effect of exercise on inflammatory factors in overweight and obese individuals. Previous reviews have discussed the effect of exercise training on chronic inflammation and its underlying mechanisms, arguing that exercise training can reduce chronic systemic inflammation in obese individuals through a variety of mechanisms (17, 22). However, the anti-inflammatory effect of exercise training is inseparable from the exercise type and intensity. Currently, the main exercise modalities for overweight and obese people include aerobic exercise (AE), resistance training (RT), combined aerobic and resistance training (CT), and high-intensity interval training (HIIT). A meta-analysis has shown that AE decreases the levels of C-reactive protein (CRP), TNF-α, and IL-6 (20). Moreover, studies have shown that RT and CT can improve the inflammatory status of overweight and obese individuals (11, 23–25). Another study has shown that HIIT demonstrates anti-inflammatory effects similar to those of CT, and it is an effective treatment strategy for overweight and obese people who need to improve their inflammatory status but have insufficient time (26). Most of the current meta-analyses investigating the effect of exercise training on inflammatory status in overweight and obese people focuse on children (27, 28). And most of them are pairwise meta-analyses. However, a pairwise meta-analysis cannot rank the effects of different interventions. Therefore, as to which type of exercise is the most effective in improving the inflammatory status of overweight and obese patients remains unknown.

Network meta-analysis (NMA) is a technique used to evaluate multiple interventions in a single analysis by combining direct and indirect evidence (29). NMA allows for the comparison of the relative effectiveness between any pair of interventions, as well as ranks the effectiveness of different interventions (29). Therefore, this paper aimed to conduct an NMA of existing randomized controlled trials (RCTs) in order to compare different exercises and comprehensively evaluate and rank their intervention effects on body composition [body weight (BW), body mass index (BMI), waist circumference (WC), percentage body fat (%BF)] and on inflammatory cytokines (CRP, TNF-α, IL-6, IL-10, and adiponectin) in overweight and obese individuals.

## 2 Methods

This systematic review and NMA are reported in accordance with the Preferred Reporting Items for Systematic Reviews and Meta-Analyses for Network Meta-Analyses (PRISMA-NMA) (30). The study protocol was registered in the PROSPERO International Prospective Register of Systematic Reviews (Registration number: CRD42022303165).

### 2.1 Search Strategy

We searched for articles in five electronic databases (PubMed, Cochrane, Embase, Web of Science, and EBSCO), and the retrieval period was from the date of their inception to November 2021. The literature search was performed according to the PICOS strategy, as follows: (P) Population: overweight or obese individuals; (I) Intervention: exercise; (C) Comparator: exercise intervention or no-exercise control; (O) Outcomes: body composition and inflammatory cytokine levels; and (S) Study type: RCTs. The main search terms were obesity, overweight, exercise, training, inflammation, C-reactive protein, interleukin, tumor necrosis factor, and randomized controlled trial. The reference lists of the selected articles were also searched to supplement the eligible studies. The detailed search strategy is shown in the online supplementary **Tables S1, 2**.

### 2.2 Study Selection

Guided by the inclusion and exclusion criteria, two researchers independently screened the studies using the EndNote software. Any disagreements in the process were resolved through a discussion or by consulting a third party, whenever necessary.

The inclusion criteria were as follows (1): The study must be an RCT. (2) The study subjects must be overweight or obese (BMI ≥ 25 kg/m^2^). (3) The intervention group must have adopted an exercise intervention (e.g., AE, RT, CT, or HIIT) for at least 4 weeks, whereas the controls had a non-exercise routine and maintained their previous lifestyle. The classification of exercise training is shown in **Table S3**. (4) Outcome measures included at least one inflammatory cytokine (IL-6, IL-10, CRP, TNF-α, and adiponectin). (5) The study must be published in English.

The exclusion criteria were as follows: (1) The exercise intervention was combined with diet control or other lifestyle changes. (2) The subjects had other diseases, such as diabetes and cardiovascular disease. (3) The full text or other pertinent data cannot be obtained after contacting the author. (4) The material is a conference abstract, a dissertation, or a case report. (5) Duplicate publications.

### 2.3 Data Extraction

Two reviewers independently extracted the following information: (i) lead author; (ii) year of publication; (iii) country; (iv) sample size; (v) mean age; (vi) mean baseline; (vii) follow-up BW, BMI, %BF, WC, and IL-6, IL-10, CRP, TNF-α, and adiponectin levels; and (viii) details of the exercise intervention (type, frequency, duration, and intensity).

### 2.4 Risk of Bias of the Individual Studies

Two authors independently assessed the risk of bias (ROB) of the included studies using the Cochrane Risk of Bias Tool (31), which covers seven domains: (i) randomized sequence generation, (ii) treatment allocation concealment, (iii) blinding of participants and personnel, (iv) blinding of outcome assessment, (v) incomplete outcome data, (vi) selective reporting, and (vii) other sources of bias. For each source of bias, the studies were classified as having a low, high, or unclear risk (if reporting was insufficient to allow for the assessment of a particular domain). If there were any disagreements, a third party will be consulted for discussion and decision.

### 2.5 Statistical Analysis

We used Review Manager 5.3 for the pairwise meta-analysis. For the NMA, we used the “mvmeta” and “network” packages in Stata 16.0. In this study, the outcome indicators were the continuous variables; standardized mean difference (SMD) and 95% confidence interval (CI) were used as effect indicators. If a study involves more than one intervention groups adopting activities that fall within the same type of exercise training (e.g., stair exercise and downstairs exercise are classified as AE), the data for those intervention groups were pooled. The heterogeneity (I^2^) and P values for the direct comparison of the exercise patterns for the intervention group with those of the control group were obtained through pairwise meta-analysis, and then NMA was carried out. The relationship between exercise interventions is presented using a network diagram. In the network geometry, the dot size represents the sample size, and the line connecting the dots indicates that direct comparison studies involving two exercise modes do exist (32). The greater the number of direct comparison studies between two interventions, the thicker the connecting line will be; otherwise, the thinner it becomes (32). If there is no connecting line between two motion modes, NMA was used for indirect comparison. First, the inconsistency factors (IF) and their 95% CI were calculated to evaluate the consistency of each closed loop; consistency is indicated by the lower limit of 95% CI being equal to 0 (33). Then, the inconsistency model is used to test for inconsistency; if P>0.05, the inconsistency is not significant, and thus the consistency model is used for analysis (32). At the same time, the node-splitting method is used to check the local inconsistency; if P > 0.05, the result is credible (34). The area under the cumulative ranking probability diagram (SUCRA) was used to rank and compare the effects of various exercise training interventions (35). SUCRA values range from 0 to 100%. The higher SUCRA values, and the closer to 100%, the higher the likelihood that a therapy is in the top rank or one of the top ranks (36). Thus, higher SUCRA values indicate better effects of an exercise intervention. Finally, the risk of publication bias was evaluated by using a correction comparison funnel. We also performed subgroup analyses and sensitivity analyses to explore the reasons for heterogeneity in pairwise meta-analyses.

## 3 Results

### 3.1 Literature Selection

**Figure 1:**
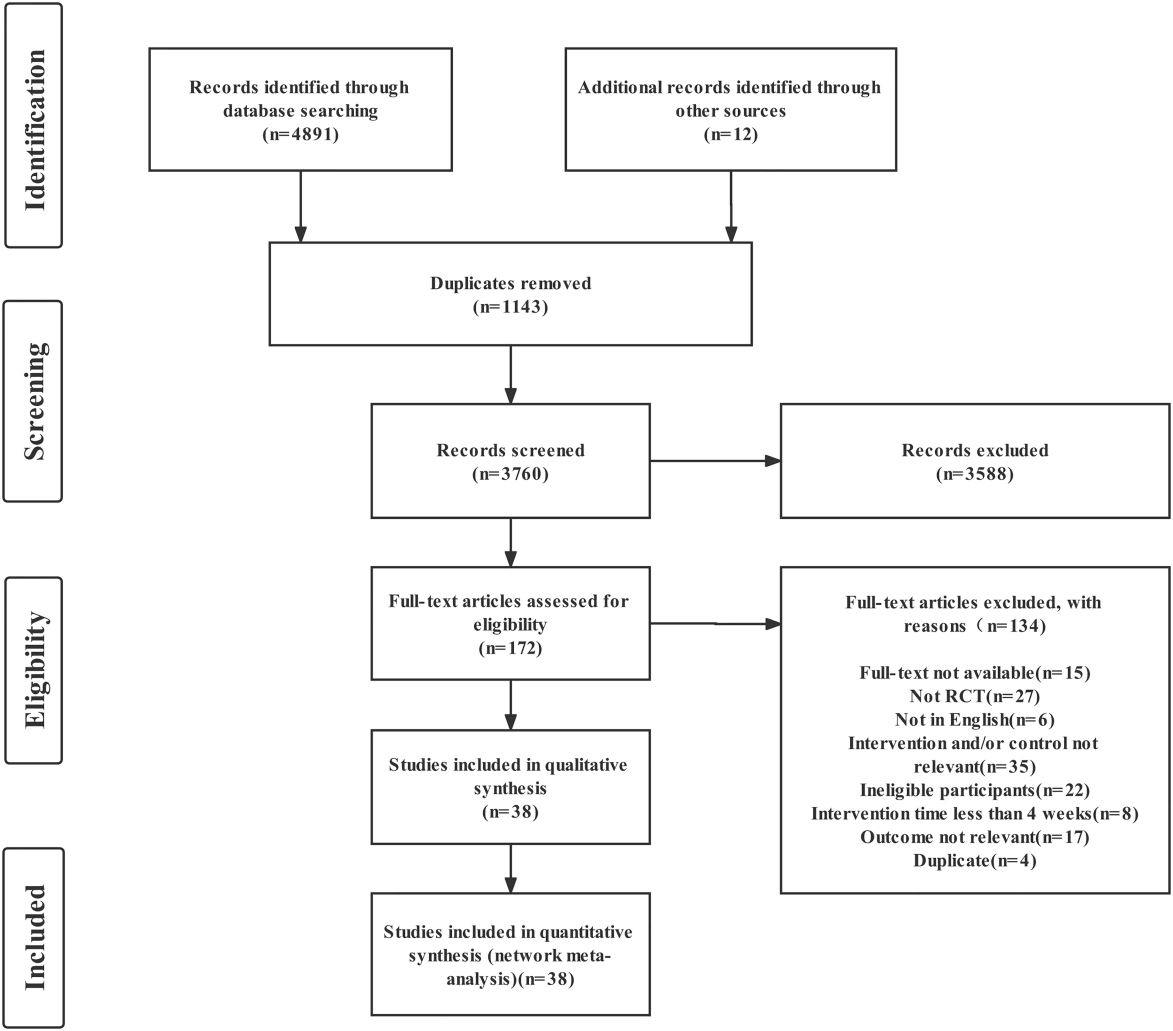
PRISMA flow diagram.

A total of 4903 studies were identified in the abovementioned databases and in other sources, and 3760 articles were left after the duplicates were removed. After the titles and abstracts were screened, 3588 articles were excluded. Finally, after the full texts were read, 38 RCTs were included in the NMA. The detailed process for the study search and selection is presented in **Figure 1**.

### 3.2 Characteristics of the Included Studies

**Figure 2:**
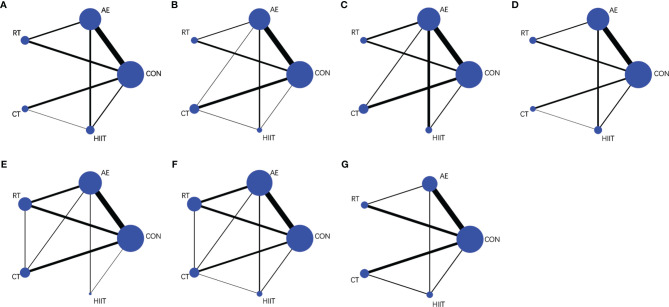
Net graphs for **(A)** body weight, **(B)** body mass index, **(C)** waist circumference, **(D)** percentage body fat, **(E)** tumor necrosis factor-alpha, **(F)** interleukin-6, and **(G)** adiponectin. AE, aerobic exercise; RT, resistance training; CT, combined aerobic and resistance training; HIIT, high-intensity interval training; CON, control group. The size of the nodes represents the number of participants in an intervention, and the thickness of lines between interventions represents the number of studies that compare them.

The basic characteristics of the included studies (n=38) are shown in **Table S4** (11, 23–26, 37–61). A total of 1317 subjects were included in this study, that is, 868 in the experimental group and 449 in the control group. There were 31 control groups and 58 exercise intervention groups. In the exercise intervention groups, the distribution of the adopted interventions was as follows: AE (n=27), RT (n=12), CT (n=11), and HIIT (n=8). Among the control groups, one group did not engage in exercises outside the school physical education class; another group did not engage in exercises but did engage in stretching, knitting, and health lectures; the other groups had no exercise. The duration of the interventions ranged from 4 weeks to 48 weeks; the majority of the interventions lasted for 12 weeks (n=19). As regards exercise frequency, three times a week was prescribed in most studies (n=29). **Figures 2A–G** shows the available direct comparisons studies for BW (30 studies), BMI (24 studies), WC (12 studies), %BF (30 studies), TNF-α (17 studies), IL-6 (23 studies), and adiponectin (14 studies).

### 3.3 ROB

Of the 38 RCTs, 7 reported the generation of random sequences, and the rest only mentioned random assignment. Due to the nature of the interventions, none of the included studies met the criteria for double blinding of the subjects and implementers. Nevertheless, all studies met the criteria for the blinding of outcome indicators, and they showed good data integrity as well as avoided selective reporting. In four studies, attrition was high, which may affect the integrity of data. Three studies possibly have other biases, and the rest did not have other biases. Details about the ROB are shown in **Figure S1**.

### 3.4 Pairwise Meta-Analysis and NMA

#### 3.4.1 BW

**Figure 3:**
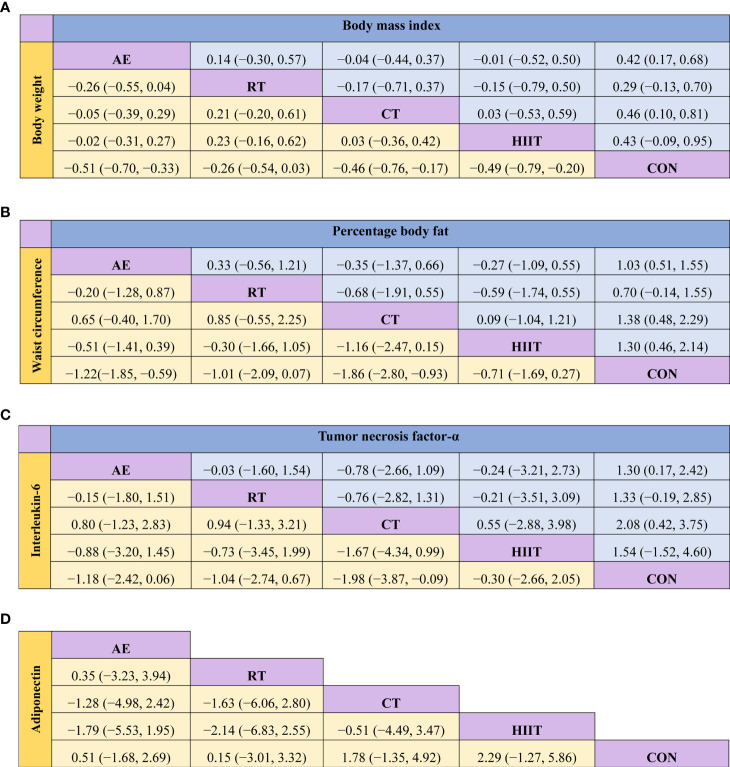
Matrix of the network meta-analysis results of **(A)** body weight and body mass index, **(B)** waist circumference and percentage body fat, **(C)** interleukin-6 and tumor necrosis factor-alpha, and **(D)** adiponectin. AE, aerobic exercise; RT, resistance training; CT, combined aerobic and resistance training; HIIT, high-intensity interval training; CON, control group. Each cell shows the SMD, along with the 95% CI.

The pairwise meta-analysis results demonstrated that exercise effectively reduced the BW in the intervention groups relative to that in the control group (SMD=−0.48; 95% CI: −0.69, −0.27; P<0.0001; I^2^ = 53%; studies: n=25; **Table S5**). The results of the consistency analysis based on the NMA indicated that, compared with the control group, the intervention groups that adopted AE (SMD=−0.51; 95% CI: −0.70, −0.33; P<0.05), CT (SMD=−0.46, 95% CI: −0.76, −0.17; P<0.05), and HIIT (SMD=−0.49; 95% CI: −0.79, −0.20; P<0.05) showed a significantly reduced BW. No significant difference in BW was observed between the control group and the RT group (SMD=−0.26; 95% CI: −0.54, 0.03; P>0.05) (**Figure 3**, **Figure S2**). The SUCRA probability sorting result showed that AE (SUCRA=78.3) had the highest probability of being the best exercise intervention for weight loss, whereas RT (SUCRA=31.9) is most likely the least effective exercise intervention (**Table S6**, **Figure S3**).

#### 3.4.2 BMI

The pairwise meta-analysis results indicated that exercise intervention effectively reduced the BMI in the intervention group relative to that in the control group (SMD=−0.41; 95% CI: −0.68, −0.14; P=0.003; I^2^ = 65%; studies: n=20; **Table S5**). The results of the consistency analysis based on the NMA showed that, compared with the control group, the intervention groups that adopted AE (SMD=−0.42; 95% CI: −0.68, −0.17; P<0.05) and CT (SMD=−0.46; 95% CI: −0.81, −0.10; P<0.05) showed a significantly reduced BMI. No significant difference in BMI was observed among the HIIT (SMD=−0.43; 95% CI: −0.95, 0.09; P>0.05), RT (SMD=−0.29; 95% CI: −0.70, 0.13; P>0.05), and control groups (**Figure 3**, **Figure S4**). The SUCRA probability sorting result showed that CT (SUCRA=70.7) is most likely the best exercise intervention for lowering BMI (**Table S6**, **Figure S5**).

#### 3.4.3 WC

Data from the pairwise meta-analysis showed that exercise intervention effectively reduced the WC in the intervention group relative to that in the control group (SMD=−1.22; 95% CI: −1.74, −0.70; P<0.00001; I^2^ = 81%; studies: n=9; **Table S5**). The results of the consistency analysis based on the NMA demonstrated that, compared with the control group, the groups that adopted AE (SMD=−1.22; 95% CI: −1.85, −0.59; P<0.05) and CT (SMD=−1.86; 95% CI: −2.80, −0.93; P<0.05) showed a significantly a reduced WC. No significant difference in WC was observed among the RT (SMD=−1.01; 95% CI: −2.09, 0.07; P>0.05), HIIT (SMD=−0.71; 95% CI: −1.69, 0.27; P>0.05), and control groups (**Figure 3**, **Figure S6**). The SUCRA probability sorting result showed that CT (SUCRA=93.4) is most likely the best exercise intervention for WC reduction (**Table S6**, **Figure S7**).

#### 3.4.4 %BF

The pairwise meta-analysis results revealed that the exercise interventions effectively reduced the %BF in the intervention group relative to that in the control group (SMD=−1.07; 95% CI: −1.47, −0.68; P<0.00001; I^2^ = 85%; studies: n=25; **Table S5**). The results of the consistency analysis based on the NMA showed that, compared with the control group, the groups that adopted AE (SMD=−1.03; 95% CI: −1.55, −0.51; P<0.05), CT (SMD=−1.38; 95% CI: −2.29, −0.48; P<0.05), and HIIT (SMD=−1.30; 95% CI: −2.14, −0.46; P<0.05) showed a significantly reduced %BF. No significant difference in %BF was observed between the RT (SMD=−0.70; 95% CI: −1.55, 0.14; P>0.05) and control groups (**Figure 3**, **Figure S8**). The SUCRA probability sorting result showed that CT (SUCRA=79.6) is most likely the best exercise intervention for reducing %BF, whereas the least effective exercise is most likely RT (SUCRA=36.7; **Table S6**, **Figure S9**).

#### 3.4.5 CRP

The pairwise meta-analysis results demonstrated that exercise intervention effectively reduced the CRP level in the intervention group relative to that in the control group (SMD=−0.76; 95% CI: −1.11, −0.41; P<0.0001; I^2^ = 78%; studies: n=20). In the 20 included studies, there were 12 items for AE, 5 items of RT, 5 items of CT, and 1 item of HIIT. A subgroup analysis involving three exercise modes was carried out, and the results indicated that AE (SMD=−0.43; 95% CI: −0.78, −0.09; P=0.01; I^2^ = 63%), RT (SMD=−0.77; 95% CI: −1.27, −0.27; P=0.003; I^2^ = 48%), and CT (SMD=−1.89; 95% CI: −3.33, −0.48; P=0.009; I^2^ = 92%) significantly reduced the CRP level in the intervention groups (**Table S5**).

#### 3.4.6 TNF-α

The pairwise meta-analysis showed that exercise intervention effectively reduced the TNF-α level in the intervention group relative to that in the control group (SMD=−1.36; 95% CI: −1.90, −0.82; P<0.00001; I^2^ = 91%; studies: n=22; **Table S5**). The results of the consistency analysis based on the NMA revealed that, compared with the control group, the groups that adopted CT (SMD=−2.08; 95% CI: −3.75, −0.42; P<0.05) and AE (SMD=−1.30; 95% CI: −2.42, −0.17; P<0.05) showed significantly reduced the TNF-α level. No significant difference was observed among the RT (SMD=−1.33; 95% CI: −2.85, 0.19; P>0.05), HIIT (SMD=−1.54; 95% CI: −4.60, 1.52; P>0.05), and control groups (**Figure 3**, **Figure S10**). The SUCRA probability sorting result showed that the different exercise methods reduced the TNF-α level in the following order: CT (SUCRA=79.4), HIIT (SUCRA=58.0), RT (SUCRA=53.9), AE (SUCRA=52.9), and CON (SUCRA=5.8) (**Table S6**, **Figure S11**).

#### 3.4.7 IL-6

The pairwise meta-analysis revealed that exercise intervention effectively reduced the IL-6 level in the intervention groups relative to that in the control group (SMD=−0.85; 95% CI: −1.42, −0.27; P=0.004; I^2^ = 91%; studies: n=19; **Table S5**). The results of the consistency analysis based on the NMA showed that, compared with the control group, the groups that adopted CT (SMD=−1.98; 95% CI: −3.87, −0.09; P<0.05) showed significantly reduced IL-6 level. No significant difference in IL-6 level was observed among the AE (SMD=−1.18; 95% CI: −2.42, 0.06; P>0.05), RT (SMD=−1.04; 95% CI: −2.74, 0.67; P>0.05), HIIT (SMD=−0.30; 95% CI: −2.66, 2.05; P>0.05), and control groups (**Figure 3**, **Figure S12**). The SUCRA probability sorting result showed that CT (SUCRA=86.4) is most likely the best exercise intervention for reducing IL-6 (**Table S6**, **Figure S13**).

#### 3.4.8 IL-10

The 8 included studies consisted of 8 control groups, 6 AE groups, 2 RT groups, and 2 CT groups. The pairwise meta-analysis presented evidence that exercise intervention effectively improved the IL-10 level in the intervention groups relative to that in the control (SMD=2.96; 95% CI: 1.39, 4.53; P=0.0002; I^2^ = 96%; **Table S5**).

#### 3.4.9 Adiponectin

The pairwise meta-analysis indicated that the adiponectin level in the exercise intervention group did not significantly differ from that in the control group (SMD=0.52; 95% CI: −0.11, 1.15; P=0.11; I^2^ = 88%; studies: n=12; **Table S5**). The results of the consistency analysis based on the NMA showed that the adiponectin level did not significant differ among the AE (SMD=0.51; 95% CI: −1.68, 2.69; P>0.05), RT (SMD=0.15; 95% CI: −3.01, 3.32; P>0.05), CT (SMD=1.78; 95% CI: −1.35, 4.92; P>0.05), HIIT (SMD=2.29; 95% CI: −1.27, 5.86; P>0.05), and control groups (**Figure 3**, **Figure S14**).

### 3.5 Inconsistency

The 95% CI for the inconsistency factor value for the closed loop involved in each index contains 0, indicating a good consistency for each closed loop; thus, the direct comparison evidence and indirect comparison evidence are quite consistent, and there is little difference in the impact of the results on the entire NMA (**Figures S15-21**). All of the inconsistency models showed that the P values were >0.05, indicating that there was no inconsistency. Therefore, the consistency model was used for analysis. Finally, the node splitting method shows that there is no inconsistency between the direct and indirect evidence.

### 3.6 Publication Bias or Small Sample Effect Test

The indexes involved in the study were tested for publication bias (**Figures S22-28**). The indexes for WC and TNF-α were asymmetric in the funnel plots, suggesting that there was a certain publication bias or small sample effect, which may have had a certain impact on the results of the corresponding indexes. The funnel plots for the other indicators were basically symmetrical, suggesting that there is a low possibility of publication bias or a small sample effect in the current study.

### 3.7 Sensitivity Analyses of Pairwise Meta-Analysis

In order to test whether the results of the paired meta-analysis are stable and reliable, we performed sensitivity analyses for BW, BMI, WC, %BF, CRP, TNF-α, IL-6, IL-10, and adiponectin. Sensitivity analysis showed that the overall results were solid and stable after the removal of each study (**Figures S29-37**).

## 4 Discussion

This systematic review and NMA compared the effects of different exercise interventions on body composition and inflammatory cytokine levels in overweight and obese individuals. This study included 38 RCTs that adopted four exercise interventions and with a total sample size of 1353. The results confirmed the beneficial effects of exercise interventions on body composition and inflammatory status of overweight and obese individuals. Furthermore, CT is most likely the best exercise intervention for improving body composition (BMI, WC, and %BF) and inflammatory cytokine levels (IL-6 and TNF-α) for this population.

### 4.1 Effect of Exercise Training on Anthropometric Outcomes in Overweight and Obese Individuals

The results showed that exercise can effectively reduce the BW, BMI, WC, and %BF of obese patients, consistent with the results of a previous pairwise meta-analysis (62). Using NMA, we observed that AE had a better effect on weight loss, whereas CT demonstrated greater effectiveness in reducing BMI, WC, and %BF.

The AHA/ACC/TOS Guidelines for the management of overweight and obesity in adults argue that a sustained weight loss of 3%–5% is likely to reduce obesity-related complications and offers greater benefits resulting from greater weight losses (63). Our finding showed that AE, CT, and HIIT could effectively reduce the weight of overweight and obese patients, and AE is possibly the most effective exercise intervention. A previous NMA showed that long-term adherence to a regular moderate-to-vigorous AE can significantly reduce BW compared with having a no-exercise lifestyle (15). Another meta-analysis revealed that 12 weeks to 12 months of AE can moderately reduce weight and can lower the risk of cardiovascular diseases (64). Consistent with previous findings, we also found that resistance training alone is not ideal for weight loss (16). The possible main reason is that RT alone is more helpful in maintaining or even increasing lean body mass. Therefore, RT is not useless for overweight and obese people. A study has shown that adding RT to caloric restriction can almost completely prevent the loss of lean body mass caused by caloric restriction, which is especially important in overweight and obese older adults (65).

Apart from weight, we also explored BMI, BF%, and WC. BMI is a powerful predictor of overall mortality, but it has limitations in reflecting changes in adipose tissue and lean muscle (5, 66). Meanwhile, %BF demonstrates higher specificity when considering the contribution of other tissue types to weight and body composition (67). Compared with BF%, WC reflects the status of abdominal obesity, and it is closely associated with the risk of cardiovascular diseases (5). In general, BMI and BF% reflect the degree of overall adiposity, whereas WC reflects the degree of central adiposity. Our study showed that CT will most likely exert the best effect in reducing BMI, %BF, and WC in overweight and obese individuals. An NMA investigating the effect of exercise intervention in obese patients has found that the combined exercise intervention involving AE and RT is the most promising intervention to reduce WC and %BF (16). A prospective cohort study also suggests that CT is more effective in preventing obesity (68). AE is beneficial for increasing energy and lipid utilization (69). The possible reason as to why RT can induce positive changes in body composition is that it increases skeletal muscle mass, further improving the basal metabolic rate and energy expenditure (70). Furthermore, lipolytic activation is delayed in obese individuals, and RT may play a role by upregulating adipose tissue lipolysis and by increasing energy expenditure (71, 72).

Our results demonstrated the important role of exercise intervention in obesity management and further confirmed the superiority of AE and CT over other forms of exercise in improving body composition. However, it is worth noting that dietary control cannot be ignored in obesity management. The current guidelines for medical care of obese patients point out that a structured lifestyle intervention program designed for weight loss should include healthy dietary plans, physical activities, and behavioral interventions (73). An NMA on the impact of long-term lifestyle programs on weight loss and cardiovascular risk factors in overweight/obese participants also suggests that diet combined with exercise intervention can be highly recommended for long-term obesity management, and dietary intervention has advantages over exercise intervention in anthropometric results (74). In a word, we recommend that overweight and obese people should adopt AE combined with RT as their primary form of exercise, while paying attention to caloric restriction.

### 4.2 Effect of Exercise Training on Pro-Inflammatory Cytokine Levels in Overweight and Obese Individuals

IL-6, TNF-α, and CRP are important pro-inflammatory factors, and their levels are elevated in people with obesity (7, 17). Studies have shown that exercise training can reduce obesity-related chronic inflammation by affecting the inflammatory mediators from various sources, including adipose tissue, muscle tissue, endothelial cells, and circulating immune cells (22). The pairwise meta-analysis showed that exercise intervention could significantly reduce the levels of IL-6, TNF-α, and CRP in overweight and obese people. Since the relevant literature on CRP indicators does not meet the requirements for an NMA, we conducted an NMA on IL-6 and TNF-α, and the results showed that CT had the highest probability of being the best exercise intervention for reducing IL-6 and TNF-α levels.

The circulating levels of IL-6 and TNF-α are directly associated with adiposity and insulin resistance (8). A previous review discussing the effects of physical activity on inflammatory mediators suggested that the combination of AE and RT is the best form of exercise to improve one’s inflammatory state, consistent with our findings (75). CT may reduce the level of inflammatory cytokines in overweight and obese individuals through the following mechanisms. Firstly, CT reduces the release of inflammatory cytokines by reducing body fat, especially visceral fat. Adipose tissue is a rich source of inflammatory cytokines, and the current results and previous findings have shown that CT is likely to be the best exercise intervention to reduce %BF and abdominal fat (76, 77). Secondly, CT is superior to AE and RT in improving muscle protein synthesis and myocellular quality (78). Moreover, exercise promotes the production of skeletal muscles and the release of muscle-derived cytokines (such as IL-6), which play significant anti-inflammatory and metabolic functions (79). Thirdly, Inflammatory monocytes (CD14+CD16+) are highly “proinflammatory”, and are potent producers of inflammatory proteins (80). Previous research indicated that CT can reduce the percentage of inflammatory monocytes in circulation (80). And another study has shown that CT can reduce CD14 + cell surface expression of toll-like receptor 4 (TLR4) and lower lipopolysaccharide-(LPS) stimulated IL-6 production (81). More high-quality studies are needed to further explore the mechanism by which CT can more effectively improve the degree of inflammation.

CRP is a chronic systemic inflammatory marker capable of predicting cardiovascular events (82). A large-scale cross-sectional study has shown that CRP is positively correlated with BMI (83). Consistent with our results, the findings of a meta-analysis indicated that exercise training reduces CRP levels and that exercise results in a greater reduction in CRP when accompanied by a reduction in BMI or %BF, further confirming the importance of improving body composition to reduce the levels of anti-inflammatory factors (84). A previous review also suggested that the unfavorable inflammatory profile related to increased adiposity can be improved during a period of weight loss (85). The results of our subgroup analysis according to exercise mode showed that AE, RT, and CT could effectively reduce CRP levels. Although the effect of exercise interventions cannot be ranked, based on the effectiveness of CT in reducing BMI and %BF, we can speculate that CT may be more effective in reducing CRP levels. Interestingly, a meta-analysis also compared the effects of exercise training and caloric restriction on inflammatory markers (86). The above study found that exercise training combined with caloric restriction could effectively improve the circulating concentrations of inflammatory factors, and caloric restriction was more effective than exercise training in reducing CRP levels (86).

We also attempted to analyze other pro-inflammatory cytokines such as IL-1β, Monocyte chemoattractant protein-1 (MCP-1), leptin, and IL-18. Their production is upregulated in the obese state, leading to the development of a chronic inflammatory state (7). However, there are few studies on these inflammatory factors, and the number cannot meet the minimum literature amount of meta-analysis.

### 4.3 Effect of Exercise Training on Anti-Inflammatory Cytokine Levels in Overweight and Obese People

IL-10 and adiponectin are significant anti-inflammatory factors. It has been reported that the circulating levels of IL-10 and adiponectin are lower in obese individuals than in normal-weight people (7, 17). Some studies have shown that exercise training can improve the levels of IL-10 and adiponectin, whereas other studies have not observed significant changes in both factors after exercise (11, 23, 59). We conducted only pairwise meta-analyses of IL-10 due to the small number of articles focusing on IL-10. The results showed that IL-10 levels significantly increased in overweight and obese individuals who adopted exercise regimens relative to that in the control group. IL-10 can promote the switch of macrophage phenotype from M1 to M2; M2 macrophages can upregulate IL-10 production, significantly enhancing the ability of IL-10 to exert anti-inflammatory effects and consequently improve insulin resistance and obesity-related complications (87). A study suggested that exercise training can increase circulating numbers of regulatory T cells, which mainly release anti-inflammatory cytokines such as IL-10 (22). Another study has shown that exercise training increases the level of muscle-derived IL-6 (88). IL-6 creates an anti-inflammatory environment by inducing anti-inflammatory cytokines such as IL-10 and IL-1Ra and inhibiting TNF-α production in adipose tissue and infiltrated macrophages (89). Furthermore, a review has shown that exercise reduces adipose tissue mass and adipocyte size, reduces macrophage infiltration, and promotes the macrophage phenotype changes from the pro-inflammatory M1 type to the anti-inflammatory M2 type, which may help increase the release of anti-inflammatory cytokines (e.g., IL-10 and adiponectin) from the adipose tissue (17). In addition, exercise training may reduce endothelial cell inflammation by increasing the number of endothelial progenitor cells, blood flow, laminar shear stress, and reducing the release of adhesion molecules, which can promote macrophages to switch from pro-inflammatory M1-type to anti-inflammatory M2-type (22). However, the results of both pairwise meta-analyses and NMA showed that adiponectin level increased after the exercise intervention, but the difference was not statistically significant.

Adiponectin is an adipose tissue-secreted factor that is negatively correlated with obesity, and its circulating levels can be used as a key marker of adipose tissue health (90). The decrease in adiponectin expression may be related to obesity or obesity-related metabolic disorders, such as insulin resistance, hyperlipidemia, and atherosclerosis (27). A meta-analysis has shown that exercise increases the level of adiponectin in overweight and obese people compared with the no-exercise regimen and the control, inconsistent with our results (91). However, the aforementioned study argues that the results for adiponectin are unreliable because they included small trials reporting extreme effects, as well as studies with high heterogeneity (91). Moreover, the authors were more inclined to speculate that exercise may have little to do with significant changes in adiponectin level (91). A review on the response and adaptation of adiponectin to acute and chronic exercise suggests that in some cases, adiponectin levels are not affected after exercise despite the reduction in body fat or BMI on the one hand; on the other hand, it seems that a training that aims to improve health and reduce weight and body fat will increase adiponectin levels at rest (92). The reasons for this discrepancy may be manifold. The most likely reason is the difference in the duration of intervention. A 24-week intervention study has reported that a moderate-to-high-intensity combined exercise increased the serum concentrations of adiponectin in middle-aged obese men (11). Another study has shown that 1 year of regular moderate-intensity RT significantly increased the level of adiponectin in overweight women (48). However, most of the studies included in the current study had a short intervention time, mostly about 12 weeks. Furthermore, the possible reasons include the initial degree of inflammation of the subjects, blood collection time, menstrual cycle, and intensity of exercise intervention, among others.

Besides IL-10 and adiponectin, we also focused on anti-inflammatory factors such as IL-4, IL-13, IL-1ra, and transforming growth factor β (TGF-β). Unfortunately, we found so little literature on these anti-inflammatory factors that a meta-analysis was impossible.

### 4.4 Strengths and Limitations

This study has several strengths. First, this paper is the first to use NMA to analyze the impact of different training modalities on inflammatory cytokines in overweight and obese individuals. Furthermore, the indicators included in this paper are relatively comprehensive and can effectively reflect the changes in body composition and inflammatory status. However, our study has some limitations. First, although the superiority of CT was demonstrated here, we did not take into account the sequence of performing AE and RT. And due to their limited number, the studies were not classified according to exercise intensity. Second, there is a high risk of heterogeneity in the pooled results of paired meta-analyses due to the differences in exercise intensity, exercise form, exercise time, exercise frequency, exercise equipment, settings, sample size, and article quality among studies. Heterogeneity was not fully resolved by sensitivity and subgroup analyses, and the results should be interpreted with caution. Third, the number of studies on different exercise interventions varied greatly; for example, 27 studies involved AE, while only 8 studies involved HIIT. Furthermore, during the literature selection process, not all existing literature could be included because the original text for some studies could not be found, and some studies used geometric means. Finally, since the included studies were all human trials, it was difficult to observe double blinding.

## 5 Conclusion

Our study demonstrated that exercise intervention could effectively improve body composition and chronic inflammatory status in overweight and obese individuals. More importantly, the results of this NMA suggested that CT is most likely the best exercise intervention for reducing BMI, WC, %BF, IL-6, and TNF-α in overweight/obese individuals. It is recommended that exercise prescriptions for overweight and obese people will involve a combination of AE and RT.

## Data Availability Statement

The original contributions presented in the study are included in the article/**Supplementary Material**. Further inquiries can be directed to the corresponding author.

## Author Contributions

All authors contributed to the article and approved the submitted version.

## Conflict of Interest

The authors declare that the research was conducted in the absence of any commercial or financial relationships that could be construed as a potential conflict of interest.

## Publisher’s Note

All claims expressed in this article are solely those of the authors and do not necessarily represent those of their affiliated organizations, or those of the publisher, the editors and the reviewers. Any product that may be evaluated in this article, or claim that may be made by its manufacturer, is not guaranteed or endorsed by the publisher.

## References

[1] Blüher M . Obesity: Global Epidemiology and Pathogenesis. Nat Rev Endocrinol (2019) 15(5):288–98. doi: 10.1038/s41574-019-0176-8 30814686

[2] González-Muniesa P Mártinez-González MA Hu FB Després JP Matsuzawa Y Loos RJF . Obesity. Nat Rev Dis Primers (2017) 3:17034. doi: 10.1038/nrdp.2017.34 28617414

[3] Di Cesare M Bentham J Stevens GA Zhou B Danaei G Lu Y Trends in Adult Body-Mass Index in 200 Countries From 1975 to 2014: A Pooled Analysis of 1698 Population-Based Measurement Studies With 19·2 Million Participants. Lancet (2016) 387(10026):1377–96. doi: 10.1016/s0140-6736(16)30054-x PMC7615134 27115820

[4] Kivimäki M Strandberg T Pentti J Nyberg ST Frank P Jokela M . Body-Mass Index and Risk of Obesity-Related Complex Multimorbidity: An Observational Multicohort Study. Lancet Diabetes Endocrinol (2022) 10(4):253–63. doi: 10.1016/s2213-8587(22)00033-x PMC8938400 35248171

[5] Haidar YM Cosman BC . Obesity Epidemiology. Clin Colon Rectal Surg (2011) 24(4):205–10. doi: 10.1055/s-0031-1295684 PMC3311487 23204935

[6] Chait A den Hartigh LJ . Adipose Tissue Distribution, Inflammation and Its Metabolic Consequences, Including Diabetes and Cardiovascular Disease. Front Cardiovasc Med (2020) 7:22. doi: 10.3389/fcvm.2020.00022 32158768 PMC7052117

[7] Ouchi N Parker JL Lugus JJ Walsh K . Adipokines in Inflammation and Metabolic Disease. Nat Rev Immunol (2011) 11(2):85–97. doi: 10.1038/nri2921 21252989 PMC3518031

[8] Fantuzzi G . Adipose Tissue, Adipokines, and Inflammation. J Allergy Clin Immunol (2005) 115(5):911–9. doi: 10.1016/j.jaci.2005.02.023 15867843

[9] Lumeng CN Bodzin JL Saltiel AR . Obesity Induces a Phenotypic Switch in Adipose Tissue Macrophage Polarization. J Clin Invest (2007) 117(1):175–84. doi: 10.1172/jci29881 PMC1716210 17200717

[10] Nguyen MT Favelyukis S Nguyen AK Reichart D Scott PA Jenn A . A Subpopulation of Macrophages Infiltrates Hypertrophic Adipose Tissue and Is Activated by Free Fatty Acids *Via* Toll-Like Receptors 2 and 4 and Jnk-Dependent Pathways. J Biol Chem (2007) 282(48):35279–92. doi: 10.1074/jbc.M706762200 17916553

[11] Brunelli DT Chacon-Mikahil MP Gáspari AF Lopes WA Bonganha V Bonfante IL . Combined Training Reduces Subclinical Inflammation in Obese Middle-Age Men. Med Sci Sports Exerc (2015) 47(10):2207–15. doi: 10.1249/mss.0000000000000658 26378946

[12] Saltiel AR Olefsky JM . Inflammatory Mechanisms Linking Obesity and Metabolic Disease. J Clin Invest (2017) 127(1):1–4. doi: 10.1172/jci92035 28045402 PMC5199709

[13] Furman D Campisi J Verdin E Carrera-Bastos P Targ S Franceschi C . Chronic Inflammation in the Etiology of Disease Across the Life Span. Nat Med (2019) 25(12):1822–32. doi: 10.1038/s41591-019-0675-0 PMC7147972 31806905

[14] Bray GA Frühbeck G Ryan DH Wilding JP . Management of Obesity. Lancet (2016) 387(10031):1947–56. doi: 10.1016/s0140-6736(16)00271-3 26868660

[15] Morze J Rücker G Danielewicz A Przybyłowicz K Neuenschwander M Schlesinger S . Impact of Different Training Modalities on Anthropometric Outcomes in Patients With Obesity: A Systematic Review and Network Meta-Analysis. Obes Rev (2021) 22(7):e13218. doi: 10.1111/obr.13218 33624411 PMC8244024

[16] O'Donoghue G Blake C Cunningham C Lennon O Perrotta C . What Exercise Prescription Is Optimal to Improve Body Composition and Cardiorespiratory Fitness in Adults Living With Obesity? A Network Meta-Analysis. Obes Rev (2021) 22(2):e13137. doi: 10.1111/obr.13137 32896055 PMC7900983

[17] Gleeson M Bishop NC Stensel DJ Lindley MR Mastana SS Nimmo MA . The Anti-Inflammatory Effects of Exercise: Mechanisms and Implications for the Prevention and Treatment of Disease. Nat Rev Immunol (2011) 11(9):607–15. doi: 10.1038/nri3041 21818123

[18] Chen X Sun X Wang C He H . Effects of Exercise on Inflammatory Cytokines in Patients With Type 2 Diabetes: A Meta-Analysis of Randomized Controlled Trials. Oxid Med Cell Longev (2020) 2020:6660557. doi: 10.1155/2020/6660557 33456672 PMC7785348

[19] Alizaei Yousefabadi H Niyazi A Alaee S Fathi M Mohammad Rahimi GR . Anti-Inflammatory Effects of Exercise on Metabolic Syndrome Patients: A Systematic Review and Meta-Analysis. Biol Res Nurs (2021) 23(2):280–92. doi: 10.1177/1099800420958068 32938197

[20] Zheng G Qiu P Xia R Lin H Ye B Tao J . Effect of Aerobic Exercise on Inflammatory Markers in Healthy Middle-Aged and Older Adults: A Systematic Review and Meta-Analysis of Randomized Controlled Trials. Front Aging Neurosci (2019) 11:98. doi: 10.3389/fnagi.2019.00098 31080412 PMC6497785

[21] Khosravi N Stoner L Farajivafa V Hanson ED . Exercise Training, Circulating Cytokine Levels and Immune Function in Cancer Survivors: A Meta-Analysis. Brain Behav Immun (2019) 81:92–104. doi: 10.1016/j.bbi.2019.08.187 31454519

[22] You T Arsenis NC Disanzo BL Lamonte MJ . Effects of Exercise Training on Chronic Inflammation in Obesity : Current Evidence and Potential Mechanisms. Sports Med (2013) 43(4):243–56. doi: 10.1007/s40279-013-0023-3 23494259

[23] Lopes WA Leite N da Silva LR Brunelli DT Gáspari AF Radominski RB . Effects of 12 Weeks of Combined Training Without Caloric Restriction on Inflammatory Markers in Overweight Girls. J Sports Sci (2016) 34(20):1902–12. doi: 10.1080/02640414.2016.1142107 26852885

[24] Kolahdouzi S Baghadam M Kani-Golzar FA Saeidi A Jabbour G Ayadi A . Progressive Circuit Resistance Training Improves Inflammatory Biomarkers and Insulin Resistance in Obese Men. Physiol Behav (2019) 205:15–21. doi: 10.1016/j.physbeh.2018.11.033 30503849

[25] Tomeleri CM Ribeiro AS Souza MF Schiavoni D Schoenfeld BJ Venturini D . Resistance Training Improves Inflammatory Level, Lipid and Glycemic Profiles in Obese Older Women: A Randomized Controlled Trial. Exp Gerontol (2016) 84:80–7. doi: 10.1016/j.exger.2016.09.005 27616162

[26] Nunes PRP Martins FM Souza AP Carneiro MAS Orsatti CL Michelin MA . Effect of High-Intensity Interval Training on Body Composition and Inflammatory Markers in Obese Postmenopausal Women: A Randomized Controlled Trial. Menopause (2019) 26(3):256–64. doi: 10.1097/gme.0000000000001207 30277921

[27] Sirico F Bianco A D'Alicandro G Castaldo C Montagnani S Spera R . Effects of Physical Exercise on Adiponectin, Leptin, and Inflammatory Markers in Childhood Obesity: Systematic Review and Meta-Analysis. Child Obes (2018) 14(4):207–17. doi: 10.1089/chi.2017.0269 PMC5994661 29762052

[28] García-Hermoso A Ceballos-Ceballos RJ Poblete-Aro CE Hackney AC Mota J Ramírez-Vélez R . Exercise, Adipokines and Pediatric Obesity: A Meta-Analysis of Randomized Controlled Trials. Int J Obes (Lond) (2017) 41(4):475–82. doi: 10.1038/ijo.2016.230 PMC5382285 28017965

[29] Rouse B Chaimani A Li T . Network Meta-Analysis: An Introduction for Clinicians. Intern Emerg Med (2017) 12(1):103–11. doi: 10.1007/s11739-016-1583-7 PMC5247317 27913917

[30] Hutton B Salanti G Caldwell DM Chaimani A Schmid CH Cameron C . The Prisma Extension Statement for Reporting of Systematic Reviews Incorporating Network Meta-Analyses of Health Care Interventions: Checklist and Explanations. Ann Intern Med (2015) 162(11):777–84. doi: 10.7326/m14-2385 26030634

[31] Higgins JP Altman DG Gøtzsche PC Jüni P Moher D Oxman AD . The Cochrane Collaboration's Tool for Assessing Risk of Bias in Randomised Trials. BMJ (2011) 343:d5928. doi: 10.1136/bmj.d5928 22008217 PMC3196245

[32] Shim S Yoon BH Shin IS Bae JM . Network Meta-Analysis: Application and Practice Using Stata. Epidemiol Health (2017) 39:e2017047. doi: 10.4178/epih.e2017047 29092392 PMC5733388

[33] Chaimani A Higgins JP Mavridis D Spyridonos P Salanti G . Graphical Tools for Network Meta-Analysis in Stata. PLoS One (2013) 8(10):e76654. doi: 10.1371/journal.pone.0076654 24098547 PMC3789683

[34] Dias S Welton NJ Caldwell DM Ades AE . Checking Consistency in Mixed Treatment Comparison Meta-Analysis. Stat Med (2010) 29(7-8):932–44. doi: 10.1002/sim.3767 20213715

[35] Salanti G Ades AE Ioannidis JP . Graphical Methods and Numerical Summaries for Presenting Results From Multiple-Treatment Meta-Analysis: An Overview and Tutorial. J Clin Epidemiol (2011) 64(2):163–71. doi: 10.1016/j.jclinepi.2010.03.016 20688472

[36] Mbuagbaw L Rochwerg B Jaeschke R Heels-Andsell D Alhazzani W Thabane L . Approaches to Interpreting and Choosing the Best Treatments in Network Meta-Analyses. Syst Rev (2017) 6(1):79. doi: 10.1186/s13643-017-0473-z 28403893 PMC5389085

[37] Paahoo A Tadibi V Behpoor N . Effectiveness of Continuous Aerobic Versus High-Intensity Interval Training on Atherosclerotic and Inflammatory Markers in Boys With Overweight/Obesity. Pediatr Exerc Sci (2021) 33(3):132–8. doi: 10.1123/pes.2020-0138 33761458

[38] Chow BC Li S Zhu X Jiao J Quach B Baker JS . Effects of Descending or Ascending Stair Exercise on Body Composition, Insulin Sensitivity, and Inflammatory Markers in Young Chinese Women With Obesity: A Randomized Controlled Trial. J Sports Sci (2021) 39(5):496–502. doi: 10.1080/02640414.2020.1829362 33012244

[39] Abd El-Kader SM Al-Jiffri OH . Impact of Aerobic Versus Resisted Exercise Training on Systemic Inflammation Biomarkers and Quality of Life Among Obese Post-Menopausal Women. Afr Health Sci (2019) 19(4):2881–91. doi: 10.4314/ahs.v19i4.10 PMC7040316 32127864

[40] Fedewa MV Hathaway ED Higgins S Forehand RL Schmidt MD Evans EM . Moderate, But Not Vigorous, Intensity Exercise Training Reduces C-Reactive Protein. Acta Cardiol (2018) 73(3):283–90. doi: 10.1080/00015385.2017.1364832 28847205

[41] Koh Y Park KS . Responses of Inflammatory Cytokines Following Moderate Intensity Walking Exercise in Overweight or Obese Individuals. J Exerc Rehabil (2017) 13(4):472–6. doi: 10.12965/jer.1735066.533 PMC5667627 29114515

[42] Chagas EFB Bonfim MR Turi BC Brondino NCM Monteiro HL . Effect of Moderate-Intensity Exercise on Inflammatory Markers Among Postmenopausal Women. J Phys Act Health (2017) 14(6):479–85. doi: 10.1123/jpah.2016-0319 28253046

[43] Park SM Kwak YS Ji JG . The Effects of Combined Exercise on Health-Related Fitness, Endotoxin, and Immune Function of Postmenopausal Women With Abdominal Obesity. J Immunol Res (2015) 2015:830567. doi: 10.1155/2015/830567 26075288 PMC4446502

[44] Ahmadizad S Avansar AS Ebrahim K Avandi M Ghasemikaram M . The Effects of Short-Term High-Intensity Interval Training Vs. Moderate-Intensity Continuous Training on Plasma Levels of Nesfatin-1 and Inflammatory Markers. Horm Mol Biol Clin Investig (2015) 21(3):165–73. doi: 10.1515/hmbci-2014-0038 25581765

[45] Ho SS Dhaliwal SS Hills AP Pal S . Effects of Chronic Exercise Training on Inflammatory Markers in Australian Overweight and Obese Individuals in a Randomized Controlled Trial. Inflammation (2013) 36(3):625–32. doi: 10.1007/s10753-012-9584-9 23250821

[46] Akbarpour M . The Effect of Aerobic Training on Serum Adiponectin and Leptin Levels and Inflammatory Markers of Coronary Heart Disease in Obese Men. Biol Sport (2013) 30(1):21–7. doi: 10.5604/20831862.1029817 PMC3944554 24744461

[47] Phillips MD Patrizi RM Cheek DJ Wooten JS Barbee JJ Mitchell JB . Resistance Training Reduces Subclinical Inflammation in Obese, Postmenopausal Women. Med Sci Sports Exerc (2012) 44(11):2099–110. doi: 10.1249/MSS.0b013e3182644984 22874536

[48] Olson TP Dengel DR Leon AS Schmitz KH . Changes in Inflammatory Biomarkers Following One-Year of Moderate Resistance Training in Overweight Women. Int J Obes (Lond) (2007) 31(6):996–1003. doi: 10.1038/sj.ijo.0803534 17299382

[49] Kim ES Im JA Kim KC Park JH Suh SH Kang ES . Improved Insulin Sensitivity and Adiponectin Level After Exercise Training in Obese Korean Youth. Obes (Silver Spring) (2007) 15(12):3023–30. doi: 10.1038/oby.2007.360 18198311

[50] Kelly AS Steinberger J Olson TP Dengel DR . In the Absence of Weight Loss, Exercise Training Does Not Improve Adipokines or Oxidative Stress in Overweight Children. Metabolism (2007) 56(7):1005–9. doi: 10.1016/j.metabol.2007.03.009 17570265

[51] Kelly AS Wetzsteon RJ Kaiser DR Steinberger J Bank AJ Dengel DR . Inflammation, Insulin, and Endothelial Function in Overweight Children and Adolescents: The Role of Exercise. J Pediatr (2004) 145(6):731–6. doi: 10.1016/j.jpeds.2004.08.004 15580192

[52] Sawyer BJ Tucker WJ Bhammar DM Ryder JR Sweazea KL Gaesser GA . Effects of High-Intensity Interval Training and Moderate-Intensity Continuous Training on Endothelial Function and Cardiometabolic Risk Markers in Obese Adults. J Appl Physiol (1985) (2016) 121(1):279–88. doi: 10.1152/japplphysiol.00024.2016 PMC4967258 27255523

[53] Vella CA Taylor K Drummer D . High-Intensity Interval and Moderate-Intensity Continuous Training Elicit Similar Enjoyment and Adherence Levels in Overweight and Obese Adults. Eur J Sport Sci (2017) 17(9):1203–11. doi: 10.1080/17461391.2017.1359679 PMC6104631 28792851

[54] Cooper JH Collins BE Adams DR Robergs RA Donges CE . Limited Effects of Endurance or Interval Training on Visceral Adipose Tissue and Systemic Inflammation in Sedentary Middle-Aged Men. J Obes (2016) 2016:2479597. doi: 10.1155/2016/2479597 27777795 PMC5061978

[55] Hornbuckle LM McKenzie MJ Whitt-Glover MC . Effects of High-Intensity Interval Training on Cardiometabolic Risk in Overweight and Obese African-American Women: A Pilot Study. Ethn Health (2018) 23(7):752–66. doi: 10.1080/13557858.2017.1294661 28277015

[56] Park W Jung WS Hong K Kim YY Kim SW Park HY . Effects of Moderate Combined Resistance- and Aerobic-Exercise for 12 Weeks on Body Composition, Cardiometabolic Risk Factors, Blood Pressure, Arterial Stiffness, and Physical Functions, Among Obese Older Men: A Pilot Study. Int J Environ Res Public Health (2020) 17(19):7233. doi: 10.3390/ijerph17197233 PMC7579509 33022918

[57] Meyer AA Kundt G Lenschow U Schuff-Werner P Kienast W . Improvement of Early Vascular Changes and Cardiovascular Risk Factors in Obese Children After a Six-Month Exercise Program. J Am Coll Cardiol (2006) 48(9):1865–70. doi: 10.1016/j.jacc.2006.07.035 17084264

[58] Nono Nankam PA Mendham AE De Smidt MF Keswell D Olsson T Blüher M . Changes in Systemic and Subcutaneous Adipose Tissue Inflammation and Oxidative Stress in Response to Exercise Training in Obese Black African Women. J Physiol (2020) 598(3):503–15. doi: 10.1113/jp278669 31873952

[59] Auerbach P Nordby P Bendtsen LQ Mehlsen JL Basnet SK Vestergaard H . Differential Effects of Endurance Training and Weight Loss on Plasma Adiponectin Multimers and Adipose Tissue Macrophages in Younger, Moderately Overweight Men. Am J Physiol Regul Integr Comp Physiol (2013) 305(5):R490–8. doi: 10.1152/ajpregu.00575.2012 23842679

[60] Lee YH Song YW Kim HS Lee SY Jeong HS Suh SH . The Effects of an Exercise Program on Anthropometric, Metabolic, and Cardiovascular Parameters in Obese Children. Korean Circ J (2010) 40(4):179–84. doi: 10.4070/kcj.2010.40.4.179 PMC2859335 20421958

[61] Mendham AE Duffield R Marino F Coutts AJ . Small-Sided Games Training Reduces Crp, Il-6 and Leptin in Sedentary, Middle-Aged Men. Eur J Appl Physiol (2014) 114(11):2289–97. doi: 10.1007/s00421-014-2953-3 25048075

[62] Kim KB Kim K Kim C Kang SJ Kim HJ Yoon S . Effects of Exercise on the Body Composition and Lipid Profile of Individuals With Obesity: A Systematic Review and Meta-Analysis. J Obes Metab Syndr (2019) 28(4):278–94. doi: 10.7570/jomes.2019.28.4.278 PMC6939705 31909371

[63] Jensen MD Ryan DH Apovian CM Ard JD Comuzzie AG Donato KA . 2013 Aha/Acc/Tos Guideline for the Management of Overweight and Obesity in Adults: A Report of the American College of Cardiology/American Heart Association Task Force on Practice Guidelines and the Obesity Society. Circulation (2014) 129(25 Suppl 2):S102–38. doi: 10.1161/01.cir.0000437739.71477.ee PMC5819889 24222017

[64] Thorogood A Mottillo S Shimony A Filion KB Joseph L Genest J . Isolated Aerobic Exercise and Weight Loss: A Systematic Review and Meta-Analysis of Randomized Controlled Trials. Am J Med (2011) 124(8):747–55. doi: 10.1016/j.amjmed.2011.02.037 21787904

[65] Sardeli AV Komatsu TR Mori MA Gáspari AF Chacon-Mikahil MPT . Resistance Training Prevents Muscle Loss Induced by Caloric Restriction in Obese Elderly Individuals: A Systematic Review and Meta-Analysis. Nutrients (2018) 10(4):423. doi: 10.3390/nu10040423 PMC5946208 29596307

[66] Whitlock G Lewington S Sherliker P Clarke R Emberson J Halsey J . Body-Mass Index and Cause-Specific Mortality in 900 000 Adults: Collaborative Analyses of 57 Prospective Studies. Lancet (2009) 373(9669):1083–96. doi: 10.1016/s0140-6736(09)60318-4 PMC2662372 19299006

[67] Millstein RA . Measuring Outcomes in Adult Weight Loss Studies That Include Diet and Physical Activity: A Systematic Review. J Nutr Metab (2014) 2014:421423. doi: 10.1155/2014/421423 25525513 PMC4262752

[68] Brellenthin AG Lee DC Bennie JA Sui X Blair SN . Resistance Exercise, Alone and in Combination With Aerobic Exercise, and Obesity in Dallas, Texas, Us: A Prospective Cohort Study. PLoS Med (2021) 18(6):e1003687. doi: 10.1371/journal.pmed.1003687 34161329 PMC8266085

[69] Walberg JL . Aerobic Exercise and Resistance Weight-Training During Weight Reduction. Implications for Obese Persons and Athletes. Sports Med (1989) 7(6):343–56. doi: 10.2165/00007256-198907060-00001 2662322

[70] Leite RD Durigan Rde C de Souza Lino AD de Souza Campos MV Souza M Selistre-de-Araújo HS . Resistance Training May Concomitantly Benefit Body Composition, Blood Pressure and Muscle Mmp-2 Activity on the Left Ventricle of High-Fat Fed Diet Rats. Metabolism (2013) 62(10):1477–84. doi: 10.1016/j.metabol.2013.05.009 23790633

[71] Chatzinikolaou A Fatouros I Petridou A Jamurtas A Avloniti A Douroudos I . Adipose Tissue Lipolysis Is Upregulated in Lean and Obese Men During Acute Resistance Exercise. Diabetes Care (2008) 31(7):1397–9. doi: 10.2337/dc08-0072 PMC2453678 18375413

[72] Kang S Park KM Sung KY Yuan Y Lim ST . Effect of Resistance Exercise on the Lipolysis Pathway in Obese Pre- and Postmenopausal Women. J Pers Med (2021) 11(9):874. doi: 10.3390/jpm11090874 34575649 PMC8471631

[73] Garvey WT Mechanick JI Brett EM Garber AJ Hurley DL Jastreboff AM . American Association of Clinical Endocrinologists and American College of Endocrinology Comprehensive Clinical Practice Guidelines for Medical Care of Patients With Obesity. Endocr Pract (2016) 22 Suppl 3:1–203. doi: 10.4158/ep161365.Gl 27219496

[74] Schwingshackl L Dias S Hoffmann G . Impact of Long-Term Lifestyle Programmes on Weight Loss and Cardiovascular Risk Factors in Overweight/Obese Participants: A Systematic Review and Network Meta-Analysis. Syst Rev (2014) 3:130. doi: 10.1186/2046-4053-3-130 25358395 PMC4227972

[75] Nimmo MA Leggate M Viana JL King JA . The Effect of Physical Activity on Mediators of Inflammation. Diabetes Obes Metab (2013) 15 Suppl 3:51–60. doi: 10.1111/dom.12156 24003921

[76] Stout MB Justice JN Nicklas BJ Kirkland JL . Physiological Aging: Links Among Adipose Tissue Dysfunction, Diabetes, and Frailty. Physiol (Bethesda) (2017) 32(1):9–19. doi: 10.1152/physiol.00012.2016 PMC5338596 27927801

[77] Yarizadeh H Eftekhar R Anjom-Shoae J Speakman JR Djafarian K . The Effect of Aerobic and Resistance Training and Combined Exercise Modalities on Subcutaneous Abdominal Fat: A Systematic Review and Meta-Analysis of Randomized Clinical Trials. Adv Nutr (2021) 12(1):179–96. doi: 10.1093/advances/nmaa090 PMC7849939 32804997

[78] Colleluori G Aguirre L Phadnis U Fowler K Armamento-Villareal R Sun Z . Aerobic Plus Resistance Exercise in Obese Older Adults Improves Muscle Protein Synthesis and Preserves Myocellular Quality Despite Weight Loss. Cell Metab (2019) 30(2):261–73.e6. doi: 10.1016/j.cmet.2019.06.008 31279675 PMC6685749

[79] Alizadeh Pahlavani H . Exercise Therapy for People With Sarcopenic Obesity: Myokines and Adipokines as Effective Actors. Front Endocrinol (Lausanne) (2022) 13:811751. doi: 10.3389/fendo.2022.811751 35250869 PMC8892203

[80] Timmerman KL Flynn MG Coen PM Markofski MM Pence BD . Exercise Training-Induced Lowering of Inflammatory (Cd14+Cd16+) Monocytes: A Role in the Anti-Inflammatory Influence of Exercise? J Leukoc Biol (2008) 84(5):1271–8. doi: 10.1189/jlb.0408244 18664531

[81] Stewart LK Flynn MG Campbell WW Craig BA Robinson JP McFarlin BK . Influence of Exercise Training and Age on Cd14+ Cell-Surface Expression of Toll-Like Receptor 2 and 4. Brain Behav Immun (2005) 19(5):389–97. doi: 10.1016/j.bbi.2005.04.003 15963685

[82] Ridker PM Rifai N Rose L Buring JE Cook NR . Comparison of C-Reactive Protein and Low-Density Lipoprotein Cholesterol Levels in the Prediction of First Cardiovascular Events. N Engl J Med (2002) 347(20):1557–65. doi: 10.1056/NEJMoa021993 12432042

[83] Visser M Bouter LM McQuillan GM Wener MH Harris TB . Elevated C-Reactive Protein Levels in Overweight and Obese Adults. Jama (1999) 282(22):2131–5. doi: 10.1001/jama.282.22.2131 10591334

[84] Fedewa MV Hathaway ED Ward-Ritacco CL . Effect of Exercise Training on C Reactive Protein: A Systematic Review and Meta-Analysis of Randomised and Non-Randomised Controlled Trials. Br J Sports Med (2017) 51(8):670–6. doi: 10.1136/bjsports-2016-095999 27445361

[85] Forsythe LK Wallace JM Livingstone MB . Obesity and Inflammation: The Effects of Weight Loss. Nutr Res Rev (2008) 21(2):117–33. doi: 10.1017/s0954422408138732 19087366

[86] Khalafi M Symonds ME Akbari A . The Impact of Exercise Training Versus Caloric Restriction on Inflammation Markers: A Systemic Review and Meta-Analysis. Crit Rev Food Sci Nutr (2022) 62(15):4226–41. doi: 10.1080/10408398.2021.1873732 33506692

[87] Jiang N Li Y Shu T Wang J . Cytokines and Inflammation in Adipogenesis: An Updated Review. Front Med (2019) 13(3):314–29. doi: 10.1007/s11684-018-0625-0 30066061

[88] Steensberg A Fischer CP Keller C Møller K Pedersen BK . Il-6 Enhances Plasma Il-1ra, Il-10, and Cortisol in Humans. Am J Physiol Endocrinol Metab (2003) 285(2):E433–7. doi: 10.1152/ajpendo.00074.2003 12857678

[89] Szostak J Laurant P . The Forgotten Face of Regular Physical Exercise: A 'Natural' Anti-Atherogenic Activity. Clin Sci (Lond) (2011) 121(3):91–106. doi: 10.1042/cs20100520 21729002

[90] Straub LG Scherer PE . Metabolic Messengers: Adiponectin. Nat Metab (2019) 1(3):334–9. doi: 10.1038/s42255-019-0041-z PMC7357716 32661510

[91] Yu N Ruan Y Gao X Sun J . Systematic Review and Meta-Analysis of Randomized, Controlled Trials on the Effect of Exercise on Serum Leptin and Adiponectin in Overweight and Obese Individuals. Horm Metab Res (2017) 49(3):164–73. doi: 10.1055/s-0042-121605 28249299

[92] Bouassida A Chamari K Zaouali M Feki Y Zbidi A Tabka Z . Review on Leptin and Adiponectin Responses and Adaptations to Acute and Chronic Exercise. Br J Sports Med (2010) 44(9):620–30. doi: 10.1136/bjsm.2008.046151 18927166

