# Supplementary material for: Effect of Exercise Training on Body Composition and Inflammatory Cytokine Levels in Overweight and Obese Individuals: A Systematic Review and Network Meta-Analysis
Source: Front Immunol. 2022 Jun 23;13:921085. doi: 10.3389/fimmu.2022.921085 (PMC9260601; doi:10.3389/fimmu.2022.921085)
Supplement: Supplementary file 1 [file DataSheet_1.pdf]

frontiers

Supplementary Material

Content	Pages
Table S1-2. Search strategy.	2-4
Table S3. The classification of exercise training.	5
Table S4. Study characteristics.	6-8
Figure S1. Cochrane risk bias evaluation chart.	9
Table S5. The results of subgroup analyses.	10-11
Figure S2-14. Interval plot and cumulative ranking probability plots of network meta-analysis.	12-18
Table S6. Ranking of exercise interventions in order of effectiveness.	19
Figure S15-21. Local inconsistency of network meta-analysis.	20-23
Figure S22-28. Funnel plots of network meta-analysis.	23-26
Figure S29-37. Sensitivity analyses of pairwise meta-analysis	27-31

Table S1. Search strategy for Cochrane.

ID	Search	Hits
1	MeSH descriptor: [Obesity] explode all trees	15037
2	MeSH descriptor: [Overweight] explode all trees	17853
3	#1 or #2	18010
4	MeSH descriptor: [Exercise] explode all trees	26876
5	(exercises):ab,ti,kw or (training):ab,ti,kw or (trainings):ab,ti,kw or (resistance training):ab,ti,kw or (strength training):ab,ti,kw or (combined training):ab,ti,kw or (high-intensity interval training):ab,ti,kw	113811
6	#4 or #5	128651
7	MeSH descriptor: [Inflammation] explode all trees	11747
8	(inflammat):ab,ti,kw OR (inflammations):ab,ti,kw OR (inflammatory):ab,ti,kw OR (inflammatory response):ab,ti,kw OR (inflammatory medium):ab,ti,kw OR (inflammatory factor):ab,ti,kw OR (inflammatory factors):ab,ti,kw OR (inflammatory marker):ab,ti,kw OR (inflammatory markers):ab,ti,kw OR (inflammatory cytokine):ab,ti,kw OR (inflammatory cytokines):ab,ti,kw	65063
9	MeSH descriptor: [C-Reactive Protein] explode all trees	4823
10	(C Reactive Protein):ab,ti,kw OR (reactive protein):ab,ti,kw OR (CRP):ab,ti,kw	28649
11	MeSH descriptor: [Interleukins] explode all trees	6555
12	(Interleukin):ab,ti,kw OR (IL-6):ab,ti,kw OR (IL-8):ab,ti,kw	26912
13	MeSH descriptor: [Tumor Necrosis Factor-alpha] explode all trees	3173
14	(Tumor Necrosis Factor alpha):ab,ti,kw OR (tumour necrosis factor):ab,ti,kw OR (TNFalpha):ab,ti,kw OR (TNF-alpha):ab,ti,kw OR (TNF-α):ab,ti,kw	15409
15	#7 or #8 or #9 or #10 or #11 or #12 or #13 or #14	105301
16	#3 and #6 and #15	305

Table S2. Search strategy for Pubmed, Web of Science, Embase, and EBSCO.

Database	Search strategy
Pubmed (290)	(((("Obesity"[Mesh]) OR ("Overweight"[Mesh])) AND (("Exercise"[Mesh]) OR (((((((exercises[Title/Abstract]) OR (training[Title/Abstract])) OR (trainings[Title/Abstract])) OR (resistance training[Title/Abstract])) OR (strength training[Title/Abstract])) OR (combined training[Title/Abstract])) OR (high-intensity interval training[Title/Abstract])))) AND ((((("Inflammation"[Mesh]) OR (((((((((((inflammat[Title/Abstract]) OR (inflammations[Title/Abstract])) OR (inflammatory[Title/Abstract])) OR (inflammatory response[Title/Abstract])) OR (inflammatory medium[Title/Abstract])) OR (inflammatory factor[Title/Abstract])) OR (inflammatory factors[Title/Abstract])) OR (inflammatory marker[Title/Abstract])) OR (inflammatory markers[Title/Abstract])) OR (inflammatory cytokine[Title/Abstract])) OR (inflammatory cytokines[Title/Abstract]))) OR (("C-Reactive Protein"[Mesh]) OR (((C Reactive Protein[Title/Abstract]) OR (reactive protein[Title/Abstract])) OR (CRP[Title/Abstract])))) OR (("Interleukins"[Mesh]) OR (((interleukin[Title/Abstract]) OR (IL-6[Title/Abstract])) OR (IL-8[Title/Abstract])))) OR (("Tumor Necrosis Factor-alpha"[Mesh]) OR (((((Tumor Necrosis Factor alpha[Title/Abstract]) OR (tumour necrosis factor[Title/Abstract])) OR (TNFalpha[Title/Abstract])) OR (TNF-alpha[Title/Abstract])) OR (TNF-α[Title/Abstract]))))) AND (randomized controlled trial[Publication Type] OR randomized[Title/Abstract] OR placebo[Title/Abstract])
Web of Science (2756)	#1(TS=(obesity or overweight)) AND Language: (English) AND Publication Type: (Article)<br><br>#2 (TS=(exercise or exercises or training or trainings or resistance training or strength training or combined training or high-intensity interval training)) AND Language: (English) AND Publication Type: (Article)<br><br>#3(TS=(inflammation or inflammat or inflammations or inflammatory or inflammatory response or inflammatory medium or inflammatory factor or inflammatory factors or inflammatory marker or inflammatory markers or inflammatory cytokine or inflammatory cytokines or C-reactive protein or CReactiveProtein or reactive protein or CRP or interleukins or Interleukin or IL-6 or IL-8 or Tumor Necrosis Factor-alpha or Tumor Necrosis Factor alpha or tumour necrosis factor or TNFalpha or TNF-alpha or TNF-α)) AND Language: (English) AND Publication Type: (Article)<br><br>#3 AND #2 AND #1

Embase (742)	Query ('obesity'/exp OR 'overweight':ab,ti) AND ('exercise'/exp OR 'exercises':ab,ti OR 'training':ab,ti OR 'trainings':ab,ti OR 'resistance training':ab,ti OR 'strength training':ab,ti OR 'combined training':ab,ti OR 'high-intensity interval training':ab,ti) AND ('inflammation'/exp OR (('inflammat':ab,ti OR 'inflammations':ab,ti OR 'inflammatory':ab,ti OR 'inflammatory response':ab,ti OR 'inflammatory medium':ab,ti OR 'inflammatory factor':ab,ti OR 'inflammatory factors':ab,ti) AND 'inflammatory marker':ab,ti) OR 'inflammatory markers':ab,ti OR 'inflammatory cytokine':ab,ti OR 'inflammatory cytokines':ab,ti OR 'c reactive protein'/exp OR 'c-reactive protein':ab,ti OR 'reactive protein':ab,ti OR 'crp':ab,ti OR 'interleukin derivative'/exp OR 'interleukins':ab,ti OR 'interleukin':ab,ti OR 'il-6':ab,ti OR 'il-8':ab,ti OR 'tumor necrosis factor'/exp OR 'tumor necrosis factor-alpha':ab,ti OR 'tumor necrosis factor alpha':ab,ti OR 'tnfalpha':ab,ti OR 'tnf-alpha':ab,ti OR 'tnf-α':ab,ti) AND ('randomized controlled trial':ab,ti OR 'randomized':ab,ti OR 'placebo':ab,ti)
EBSCO (798)	(AB ((obesity or overweight))) AND AB ((exercise or exercises or training or trainings or resistance training or strength training or combined training or high-intensity interval training)) AND AB ((inflammation or inflammat or inflammations or inflammatory or inflammatory response or inflammatory medium or inflammatory factor or inflammatory factors or inflammatory marker or inflammatory markers or inflammatory cytokine or inflammatory cytokines or C-reactive protein or CReactiveProtein or reactive protein or CRP or interleukins or Interleukin or IL-6 or IL-8 or Tumor Necrosis Factor-alpha or Tumor Necrosis Factor alpha or tumour necrosis factor or TNFalpha or TNF-alpha or TNF-α))) AND (S2 OR S4)

Table S3. The classifications of exercise training.

**Type**	**Definition**
Aerobic exercise （AE）	Exercise training designed to improve the efficiency and capacity of the cardiorespiratory system, such as walking, basketball, soccer(1).
Resistance training （RT）	Exercise training designed to improve the strength, power, endurance, and size of skeletal muscles, such as elastic bands, weight machines(1).
Combined aerobic and resistance training （CT）	A combination of aerobic exercise and resistance training.
High-intensity interval training （HIIT）	Exercises training involves repeated short-to-long bouts of rather high-intensity exercise interspersed with recovery periods(2).

References:

1. Powell KE, Paluch AE, Blair SN. Physical Activity for Health: What Kind? How Much? How Intense? On Top of What? *Annu Rev Public Health* (2011) 32:349-65. Epub 2010/12/07. doi: 10.1146/annurev-publhealth-031210-101151.
2. Buchheit M, Laursen PB. High-Intensity Interval Training, Solutions to the Programming Puzzle: Part I: Cardiopulmonary Emphasis. *Sports Med* (2013) 43(5):313-38. Epub 2013/03/30. doi: 10.1007/s40279-013-0029-x.

Table S4. Study characteristics.

Study	Country	Sample N/F/M	Mean age (SD)	Exercise category	Intervening measure and intensity	Intervention duration (minutes per session, times per week, total weeks)	Outcome measures reported
Paahoo 2021	Iran	15/0/15	11.20 ± 0.9	CON	No exercise		BW, BMI, %BF, CRP, IL-6
		15/0/15	10.87 ± 1.1	AE	40%-70% HRR; running	45 min, 3 d/w, 12 w	
		15/0/15	11.13 ± 1.0	HIIT	100%-110% MAS； 3 sets×10 reps (with 10s active rest intervals)	45 min, 3 d/w, 12 w	
Kang 2020	Korea	20/20/0	52.50 ± 7.65	RT	55-65% 1RM	60 min, 3 d/w, 12 w	BW, BMI, %BF, IL-6
		21/21/0	56.67 ± 5.43	AE	50-60% HRR; treadmill	50 min, 3 d/w, 12 w	
Chow 2021	China	10/10/0	19.9 ± 0.9	AE	descending stair	3 d/w, 12 w	BW, BMI, %BF, TNF-α, IL-6
		11/11/0	19.7 ± 0.9	AE	ascending stair	3 d/w, 12 w	
		10/10/0	19.4 ± 0.5	CON	No exercise		
Nunes 2019	Brazil	13/13/0	62.9 ± 2.65	CT	70% 1RM for RT； 70% MHR for AE	60 min, 3 d/w, 12 w	BW, BMI, %BF, IL-6, Adiponectin
		13/13/0	62.3 ± 2.08	HIIT	>80% MHR， 10 sets × 60s; 60% MHR， 60seconds	28 min, 3 d/w, 12 w	
Kolahdouzi 2019	Iran	13/0/13	24.0 ± 4.8	CON	No exercise		BW, BMI, hs-CRP
		13/0/13	23.0±3.8	RT	1-4 weeks, 65%-75% 1RM; 4-8 weeks, 75%-85% 1RM	60 min, 3 d/w, 8 w	
Abd 2019	Saudi Arabia	28/28/0	56.42 ± 4.81	AE	60-70% HRmax; treadmill	40 min, 3 d/w, 12 w	CRP, TNF-α, IL-6
		31/31/0	54.97 ± 5.63	RT	resistance gym machines	40 min, 3 d/w, 12 w	
Shabani 2018	Iran	10/10/0	56.90 ± 4.93	CON	No exercise		BW, BMI, hs-CRP
		12/12/0	54.83 ± 4.72	CT	50%-75% 1RM for RT; 50%-80% THR for AE	90 min, 3 d/w, 8 w	
Fedewa 2018	USA	23/23/0	20.3 ± 1.6	AE	60-70% HRR; running	20-30 min, 3 d/w, 6 w	BW, %BF, CRP
		21/21/0	20.2 ± 1.8	HIIT	5-7 reps × 30s maximal effort sprints; 4-min active recovery	3 d/w, 6 w	
Koh 2017	USA	15/–/–	33.24 ± 5.67	AE	70% HRmax; treadmill	60 min, 3 d/w, 4 w	BW, BMI, %BF, CRP, TNF-α, Adiponectin
		12/–/–	32.54 ± 5.28	CON	No exercise		
Chagas 2017	Brazil	35/35/0	61.3 ± 6.4	AE	50%~60% VO2peak; walking	75 min, 3 d/w, 20 w	BW, BMI, %BF, WC, TNF-α, IL-6, IL-10
		35/35/0	59.8 ± 7.1	CON	No exercise		
Tomeleri 2016	Brazil	19/19/0	69.5 ± 4.7	CON	No exercise		%BF, CRP, TNF-α, IL-6
		19/19/0	66.8 ± 3.2	RT	3 sets × (10-15) reps maximum	45-50 min, 3 d/w, 8 w	
Shahram 2016	Iran	10/10 /0	22.4 ± 1.64	AE	60%~75% THR, running	3 d/w, 12 w	BW, BMI, %BF, TNF-α, IL-6, IL-10
		10/10 /0	22.3 ± 1.41	RT	4 sets × 12 maximal reps at 50-60% 1RM	3 d/w, 12 w	
		10/10 /0	22.77 ± 1.63	CON	No exercise		
Salamat 2016	Iran	11/0/11	24.6 ± 2.56	AE	45-50% to 75-80% HRR, running	20-33min, 3 d/w, 8 w	TNF-α, IL-6
		11/0/11	23.5 ± 3.21	RT	50% to 85% 1RM, Weekly increased 5% 1RM	3 d/w, 8 w	
		11/0/11	22.9 ± 3.34	CT	trained 3 session endurance and resistance in two weeks alternatively	3 d/w, 8 w	
		10/0/10	23.8 ± 4.11	CON	No exercise		
Lopes 2016	Brazil	17/17/0	14.6 ± 1.15	CT	RT,3 sets × (6-10) reps at 60-70% 1 RM; AE(walking/running) at 50-80% VO2peak	60min, 3 d/w, 12 w	BW, BMI, %BF, TNF-α, IL-6, CRP, IL-10
		16/16/0	14.4 ± 1.16	CON	No exercise		
Park 2015	Korea	10/10/0	57.20 ± 2.57	CT	RT(weeks1-6, 60% 1RM; weeks 7-12, 70% 1RM); AE(1-6 weeks HRR, 40-55%; weeks 7-12, 56-75% HRR)	70(40AE+30RT) min, 3 d/w, 12 w	BW, %BF, TNF-α

		10/10/0	57.20 ± 1.69	CON	No exercise		
Brunelli 2015	Brazil	17/0/17<br>13/0/13	49.29 ± 1.31<br>48.0 ± 1.72	CT<br>CON	6-10 RM for RT, 50-85% VO_2peak for AE<br>No exercise	60 min, 3 d/w, 24 w	BW, BMI, %BF, WC, CRP, TNF-α, IL-6, IL-10, Adiponectin
Ahmadizad 2015	Iran	10/0/10<br>10/0/10<br>10/0/10	25 ± 1	CON<br>HIIT<br>AE	No exercise<br>90% vVo_2max; 8 exercise intervals per session with 2-3 min of active rest (rest/exercise ratio was 2:1)<br>50%-60% VO_2max; walking/jogging	 3 d/w, 6 w<br>30-70min, 3 d/w, 6 w	BW, %BF, TNF-α, IL-6
Nikseresht 2014b	Iran	12/0/12<br>10/0/10<br>11/0/11	40.4 ± 5.2<br>39.6 ± 3.7<br>38.9 ± 4.1	RT<br>AE<br>CON	different intensities; Nonlinear Resistance Training<br>different intensities; Aerobic Interval Training (treadmill)<br>No exercise	40-65 min, 3 d/w, 12 w<br>3 d/w, 12 w	BW, %BF, WC, CRP, TNF-α, IL-6, Adiponectin
Nikseresht 2014a	Iran	10/0/10<br>12/0/12<br>12/0/12	38.9 ± 4.1<br>40.4 ± 5.2<br>39.6 ± 3.7	CON<br>RT<br>AE	No exercise<br>different intensities; Nonlinear Resistance Training<br>different intensities; Aerobic Interval Training (treadmill)	 45-65 min, 3 d/w, 12 w<br>3 d/w, 12 w	BW, WC, TNF-α, IL-10
Mezghanni 2014	Tunisia	10/10/0<br>11/11/0<br>10/10/0<br>12/12/0	25 ± 4<br>27 ± 4<br>25 ± 5<br>28 ± 5	CON<br>AE<br>AE<br>AE	No exercise<br>50% HRR; walking(Week 1-2, 20-25 min; Week 3-6, 30-55 min; Week 7-12, 55-60 min)<br>75% HRR; walking(Week 1-2, 20-25 min; Week 3-6, 30-55 min; Week 7-12, 55-60 min)<br>50-75% HRR; walking(Week 1-2, 20-25 min; Week 3-6, 30-55 min; Week 7-12, 55-60 min)	 20-60 min, 5 d/w ,12 w<br>20-60 min, 5 d/w ,12 w<br>20-60 min, 5 d/w ,12 w	BW, %BF, WC, CRP
Mendham 2014	Australia	10/0/10<br>11/0/11<br>11/0/11	46.8 ± 6.6<br>49.5 ± 6.6<br>49.2 ± 7	AE<br>AE<br>CON	intensity:unclear; small-sided games<br>intensity:unclear; cycling training<br>No exercise	40-50 min, 3 d/w, 8 w<br>40-50 min, 3 d/w, 8 w	BW, BMI, %BF, CRP, TNF-α, IL-6, IL-10, Adiponectin
Ho 2013	Australia	16/15/1<br>15/12/3<br>16/13/3<br>17/14/3	52 ± 6.5<br>55 ± 4.5<br>52 ± 4<br>53 ± 5.25	CON<br>AE<br>RT<br>CT	No exercise<br>60% HRR; treadmill<br>10-RM; 4 sets × (8-12) reps<br>10-RM for RT, 60% HRR for AE (two sets of the above exercises)	 30 min, 5 d/w, 12 w<br>30 min, 5 d/w, 12 w<br>30 min, 5 d/w, 12 w	TNF-α, IL-6
Akbarpour 2013	Iran	30/0/30<br>30/0/30	23.2 ± 2.5<br>22.7 ± 2.7	AE<br>CON	75 ~ 85% HRmax, running<br>No exercise	25-40 min, 3 d/w, 12 w	%BF, CRP, TNF-α, IL-6, Adiponectin
Phillips 2012	USA	11/11/0<br>12/12/0	64.8 ± 2.4<br>66.4 ± 2.8	RT<br>CON	8RM; 3 sets × 10 reps<br>stretching, knitting, health lectures	3 d/w, 12 w<br>2 d/w, 12 w	BW, BMI, %BF, CRP, TNF-α, Adiponectin
Olson 2007	USA	16/16/0<br>12/12/0	39 ± 5<br>40 ± 5	RT<br>CON	moderate-intensity; 3 sets × (8-10) reps<br>No exercise	2 d/w, 48 w	BW, BMI, %BF, CRP, IL-6, Adiponectin
Kim 2007	Korea	14/0/14<br>12/0/12	17 ± 0.11	AE<br>CON	week1-3, 60jumps/min; week4-6, 90jumps/min;jump rope and regular physical education class<br>regular physical education class	40min, 5 d/w, 6 w	BW, BMI, %BF, WC, TNF-α, IL-6
Kelly 2007	USA	9/5/4<br>10/6/4	10.8 ± 0.67<br>11.0 ± 0.71	AE<br>CON	50% -60% to 70%-80% VO_2max; stationary cycling<br>No exercise	30-50 min, 4 d/w, 8 w	BW, BMI, %BF, CRP, TNF-α, IL-6, Adiponectin
Kelly 2004	USA	10/5/5<br>10/6/4	11.0 ± 0.63<br>11.0 ± 0.71	AE<br>CON	50%-60% to 70%-80% VO_2max; stationary cycling<br>No exercise	30-50 min, 4 d/w, 8 w	BW, BMI, %BF, CRP
Sawyer 2016	USA	9/5/4<br>9/4/5	34.8 ± 7.7<br>35.6 ± 8.9	AE<br>HIIT	70 -75% HRmax; moderate-intensity continuous training<br>10 × 1 min, 90-95% HRmax; 1-min active recovery	40 min, 3 d/w, 8 w<br>29 min, 3 d/w, 8 w	BW, BMI, %BF, WC, hs-CRP
Vella 2017	USA	9/4/5	28.9 ± 8.1	AE	55-59% HRR; moderate-intensity continuous training	30 min, 4 d/w, 8 w	WC, CRP, TNF-α, IL-6, Adiponectin

		8/6/2	23.1 ± 6.6	HIIT	ten 1-min bouts at 75-80% HRR, separated by ten 1-min recovery bouts at 35-40% HRR	30 min, 4 d/w, 8 w	
Cooper2016	Australia	15/0/15<br>14/0/14	51.1 ± 5.7<br>51.2 ± 7	AE<br>CON	80% HRmax(50min/session for Wks 1-6 and increased to 60min/session for Wks 7-12); cycling<br>No exercise	50-60 min, 3 d/w, 12 w	BW, %BF, CRP, TNF-α, IL-6, IL-10
Hornbuckle 2017	USA	11/11/0<br>16/16/0	28.3 ± 6.1<br>32.1 ± 7.0	AE<br>HIIT	60-70% HRmax; treadmill walking<br>treadmill HIIT alternating 3 min at 60-70% of HRmax with 1 min at 80-90% of HRmax	3 d/w, 16 w<br>32 min, 3 d/w, 16 w	BW, BMI, %BF, WC, CRP
Park 2020	Korea	10/0/10<br>10/0/10	68.5 ± 0.9<br>69.1 ± 0.9	CON<br>CT	No exercise<br>60%-70% 1 RM for RT(elastic-band resistance training), 60%-70% HRmax for AE(bicycle,treadmill)	 90-120 min, 3 d/w, 12 w	BW, BMI, %BF, TNF-α, IL-6
Meyer 2006	Germany	33/16/17<br>34/17/17	13.7 ± 2.1<br>14.1 ± 2.4	AE<br>CON	on Mondays, swimming and aqua aerobic training (60 min); Wednesdays, sports games (90 min); and Fridays,walking (60 min)<br>No exercise	60 min, 3 d/w, 24 w	BMI, %BF, CRP
Nono 2020	South Africa	20/20/0<br>15/15/0	20-35	CT<br>CON	75-80% HRpeak for AE (dance, running, skipping and stepping); 60% to 70% HRpeak for RT<br>No exercise	40-60 min, 4 d/w, 12 w	BW, BMI, %BF, WC, CRP, TNF-α, Adiponectin
Auerbach 2013	Denmark	12/0/12<br>12/0/12	20-40	AE<br>CON	3-4 days per week the training was intense (~85% HRR); the intensity was moderate (~65% of HRR) for the remaining sessions<br>No exercise	7 d/w, 12 w	BW, BMI, %BF, CRP, TNF-α, IL-6, IL-10, Adiponectin
Lee 2010	Korea	20/–/–<br>16/–/–<br>18/–/–	12-14	CT<br>AE<br>CON	moderate (70-80% of maximum strength); each time including two circuit weight training routines and one aerobic exercise routine.<br>70-90% HRmax; items that can trigger the interest of children, such as soccer, basketball, football<br>No exercise	60min, 3 d/w, 10 w<br>60min, 3 d/w, 10 w	BMI, WC, hs-CRP
Racil 2016	Tunisia	23/23/0<br>19/19/0	16.6 ± 0.9<br>16.9 ± 1.0	HIIT<br>CON	2 blocks per session of 6-8 bouts of 30-s runs at 100% velocity at peak oxygen uptake: vVO_2peak , with 30-s active recovery between bouts at 50% vVO_2peak;<br>No exercise	3 d/w, 12 w	BW, %BF, WC, Adiponectin

Abbreviations: AE, aerobic exercise; RT, resistance training; CT, combined aerobic and resistance training; HIIT, high-intensity interval training; CON, control group; BW, body weight; BMI, body mass index; WC, waist circumference; %BF, percentage body fat; CRP, C-reactive protein; hsCRP, high sensitivity C-reactive protein; TNF-α, tumor necrosis factor-α; IL, interleukin; HRR, heart rate reserve; HRmax, maximal heart rate; HRpeak, peak heart rate; VO2 peak, Peak oxygen uptake; reps, repetitions; RM, repetition maximum.

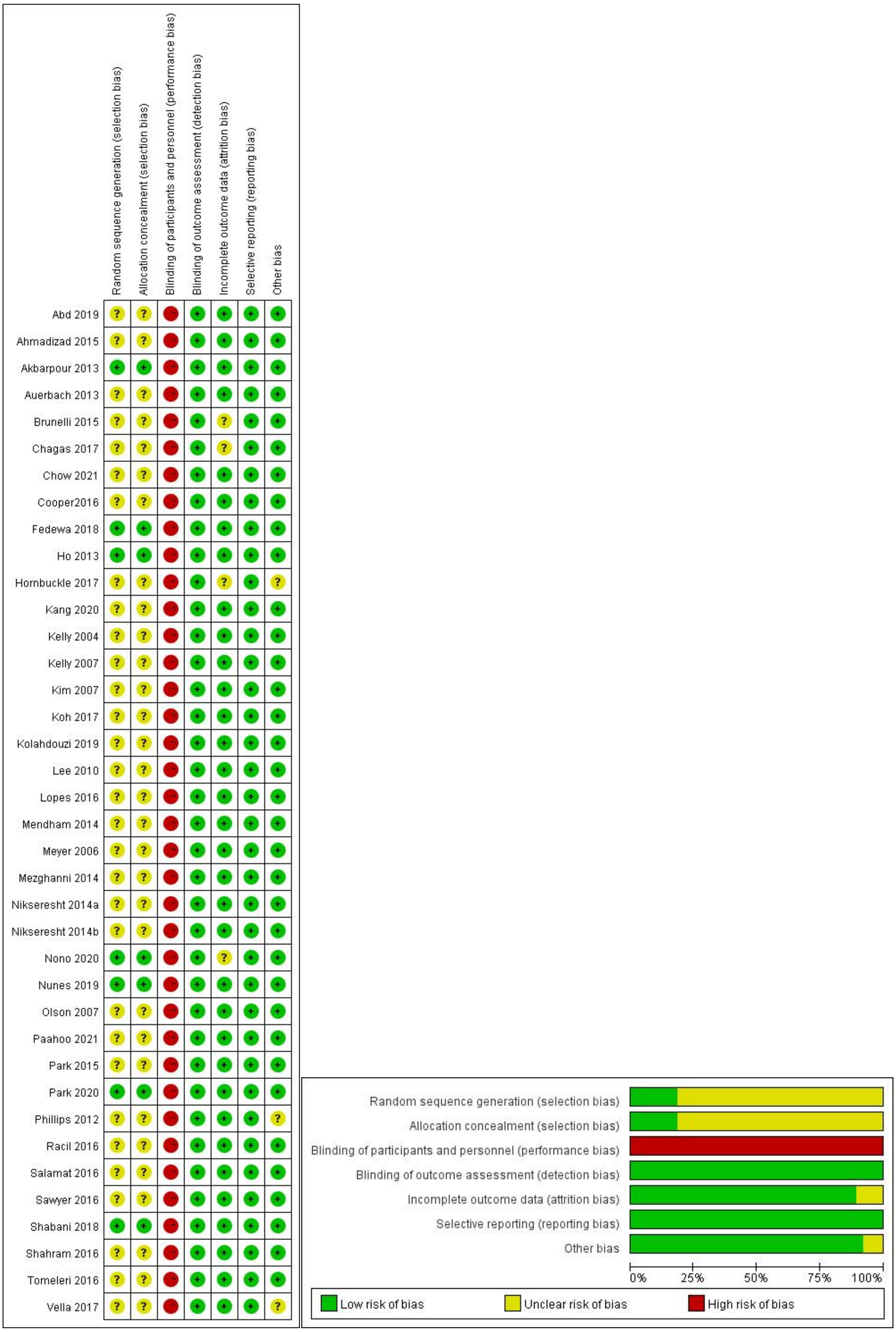

Figure S1. Cochrane risk bias evaluation chart.

Table S5. The results of subgroup analyses.

Analysis	Body weight				Body mass index				Waist circumference				percentage body fat			
	Studies	SMD (95% CI)	P-value	I^2 (%)	Studies	SMD (95% CI)	P-value	I^2 (%)	Studies	SMD (95% CI)	P-value	I^2 (%)	Studies	SMD (95% CI)	P-value	I^2 (%)
**Exercise**	25	−0.48 (−0.69, −0.27)	**<0.001**	53	20	−0.41 (−0.68, −0.14)	**0.003**	65	9	−1.22 (−1.74, −0.70)	**<0.001**	81	25	−1.07 (−1.47, −0.68)	**<0.001**	85
AE	15	−0.62 (−1.01, −0.22)	**0.002**	72	12	−0.48 (−0.92, −0.04)	**0.03**	75	6	−1.15 (−1.90, −0.40)	**0.003**	82	16	−1.09 (−1.70, −0.49)	**<0.001**	89
RT	6	−0.29 (−0.70, 0.13)	0.17	33	4	−0.22 (−0.64, 0.21)	0.32	9	2	−1.08 (−1.71, −0.44)	**0.001**	0	5	−0.66 (−1.35, 0.03)	0.06	71
CT	6	−0.46 (−0.77, −0.14)	**0.005**	0	6	−0.48 (−1.01, 0.05)	0.08	66	3	−1.80 (−3.45, −0.15)	**0.03**	91	5	−1.48 (−2.54, −0.42)	**0.006**	86
HIIT	3	−0.41 (−0.82, 0.01)	0.05	0	1	−0.27 (−0.99, 0.45)	0.47	−	1	−0.41 (−1.02, 0.20)	0.19	−	3	−1.21 (−1.80, −0.62)	**<0.001**	39
**Duration**	25	−0.48 (−0.69, −0.27)	**<0.001**	53	20	−0.41 (−0.68, −0.14)	**0.003**	65	9	−1.22 (−1.74, −0.70)	**<0.001**	81	25	−1.07 (−1.47, −0.68)	**<0.001**	85
4-8 weeks	8	−0.24 (−0.51, 0.03)	0.09	0	7	−0.31 (−0.63, 0.01)	0.06	7	1	−0.32 (−1.10, 0.45)	0.41	−	7	−0.40 (−0.68, −0.11)	**0.006**	0
9-12 weeks	14	−0.65 (−0.98, −0.32)	**<0.001**	66	9	−0.51 (−1.01, −0.01)	**0.04**	78	6	−1.16 (−1.77, −0.54)	**<0.001**	81	14	−1.23 (−1.72, −0.73)	**<0.001**	83
>12 weeks	3	−0.40 (−1.02, 0.23)	0.21	64	4	−0.51 (−0.89, −0.12)	**0.01**	40	2	−1.86 (−2.34, −1.38)	**<0.001**	0	4	−1.79 (−3.71, 0.12)	0.07	96
**Age**	25	−0.48 (−0.69, −0.27)	**<0.001**	53	20	−0.41 (−0.68, −0.14)	**0.003**	65	9	−1.22 (−1.74, −0.70)	**<0.001**	81	25	−1.07 (−1.47, −0.68)	**<0.001**	85
<18 years	6	−0.24 (−0.52, 0.04)	0.09	0	7	−0.16 (−0.44, 0.12)	0.26	30	3	−0.27 (−0.61, 0.06)	0.11	0	7	−0.48 (−0.89, −0.07)	**0.02**	62
18-45 years	15	−0.62 (−0.94, −0.30)	**<0.001**	66	8	−0.71 (−1.36, −0.05)	**0.04**	81	4	−1.67 (−2.32, −1.02)	**<0.001**	66	13	−1.61 (−2.22, −0.99)	**<0.001**	87
>45 years	4	−0.41 (−0.82, 0.01)	0.06	0	5	−0.63 (−0.95, −0.31)	**<0.001**	0	2	−1.86 (−2.34, −1.38)	**<0.001**	0	5	−0.56 (−1.38, 0.27)	0.18	79

Table S5. Continued.

Analysis	C-reactive protein				Tumor necrosis factor-α				Interleukin-6			
	Studies	SMD (95% CI)	P-value	I^2 (%)	Studies	SMD (95% CI)	P-value	I^2 (%)	Studies	SMD (95% CI)	P-value	I^2 (%)
**Exercise**	20	−0.76 (−1.11, −0.41)	**<0.001**	78	22	−1.36 (−1.90, −0.82)	**<0.001**	91	19	−0.85 (−1.42, −0.27)	**0.004**	91
AE	12	−0.43 (−0.78, −0.09)	**0.01**	63	15	−1.14 (−1.92, −0.36)	**0.004**	92	14	−0.58 (−1.38, 0.21)	0.15	92
RT	5	−0.77 (−1.27, −0.27)	**0.003**	48	7	−1.63 (−2.79, −0.47)	**0.006**	90	6	−0.42 (−1.49, 0.64)	0.43	88
CT	5	−1.89 (−3.30, −0.48)	**0.009**	92	7	−1.85 (−3.18, −0.52)	**0.006**	93	5	−1.92 (−3.87, 0.03)	0.05	95
HIIT	1	−1.99 (−2.89, −1.10)	**<0.001**	−	1	−0.75 (−1.66, 0.17)	0.11	−	2	−1.28 (−2.96, 0.41)	0.14	86
**Duration**	20	−0.76 (−1.11, −0.41)	**<0.001**	78	22	−1.36 (−1.90, −0.82)	**<0.001**	91	19	−0.85 (−1.42, −0.27)	**0.004**	91
4-8 weeks	7	−0.33 (−0.74, 0.09)	0.12	47	7	−0.32 (−0.69, 0.06)	0.1	51	6	−0.42 (−0.98, 0.14)	0.14	73
9-12 weeks	10	−0.82 (−1.22, −0.42)	**<0.001**	72	13	−1.41 (−2.07, −0.76)	**<0.001**	89	10	−0.75 (−1.60, 0.11)	0.09	92
>12 weeks	3	−2.74 (−5.16, −0.32)	**0.03**	95	2	−8.67 (−14.57, −2.77)	**0.004**	91	3	−3.35 (−5.89, −0.80)	**0.01**	95
**Age**	20	−0.76 (−1.11, −0.41)	**<0.001**	78	22	−1.36 (−1.90, −0.82)	**<0.001**	91	19	−0.85 (−1.42, −0.27)	**0.004**	91
<18 years	6	−0.65 (−1.27, −0.02)	**0.04**	82	3	0.33 (−0.12, 0.79)	0.15	0	4	−0.92 (−2.16, 0.31)	0.14	90
18-45 years	9	−0.63 (−0.90, −0.36)	**<0.001**	23	11	−0.75 (−1.25, −0.25)	**0.003**	82	9	−0.74 (−1.57, 0.09)	0.08	90
>45 years	5	−1.85 (−3.31, −0.39)	**0.01**	92	8	−2.85 (−4.14, −1.56)	**<0.001**	94	6	−1.02 (−2.16, 0.11)	0.08	94

Table S5. Continued.

Analysis	Interleukin-10				Adiponectin			
	Studies	SMD (95% CI)	P-value	I^2 (%)	Studies	SMD (95% CI)	P-value	I^2 (%)
**Exercise**	8	2.96 (1.39, 4.53)	**<0.001**	96	12	0.52 (−0.11, 1.15)	0.11	88
AE	6	2.08 (0.06, 4.09)	**0.04**	96	6	0.16 (−0.62, 0.94)	0.69	83
RT	2	4.12 (−2.97, 11.21)	0.25	96	3	0.33 (−0.54, 1.21)	0.46	70
CT	2	7.17 (−7.52, 21.85)	0.34	98	3	2.77 (−0.09, 5.63)	0.06	96
HIIT	0	−	−	−	1	1.16 (0.50, 1.82)	**<0.001**	−
**Duration**	8	2.96 (1.39, 4.53)	**<0.001**	96	12	0.52 (−0.11, 1.15)	0.11	88
4-8 weeks	1	−0.11 (−0.84, 0.62)	0.77	−	3	−0.21 (−0.82, 0.40)	0.5	41
9-12 weeks	5	1.59 (0.26, 2.93)	**0.02**	92	7	−0.34 (−0.29, 0.98)	0.29	83
>12 weeks	2	9.99 (1.00, 18.98)	**0.03**	95	2	−6.01 (−4.96, 16.97)	0.28	98
**Age**	8	2.96 (1.39, 4.53)	**<0.001**	96	12	0.52 (−0.11, 1.15)	0.11	88
<18 years	1	−0.19 (−0.87, 0.50)	0.59	−	3	0.06 (−1.11, 1.24)	0.92	86
18-45 years	4	2.07 (0.37, 3.77)	**0.02**	93	7	0.35 (−0.21, 0.91)	0.21	77
>45 years	3	6.45 (1.25, 11.65)	**0.01**	98	2	5.49 (−6.52, 17.50)	0.37	98

Abbreviations: AE, aerobic exercise; RT, resistance training; CT, combined aerobic and resistance training; HIIT, high-intensity interval training; CON, control group

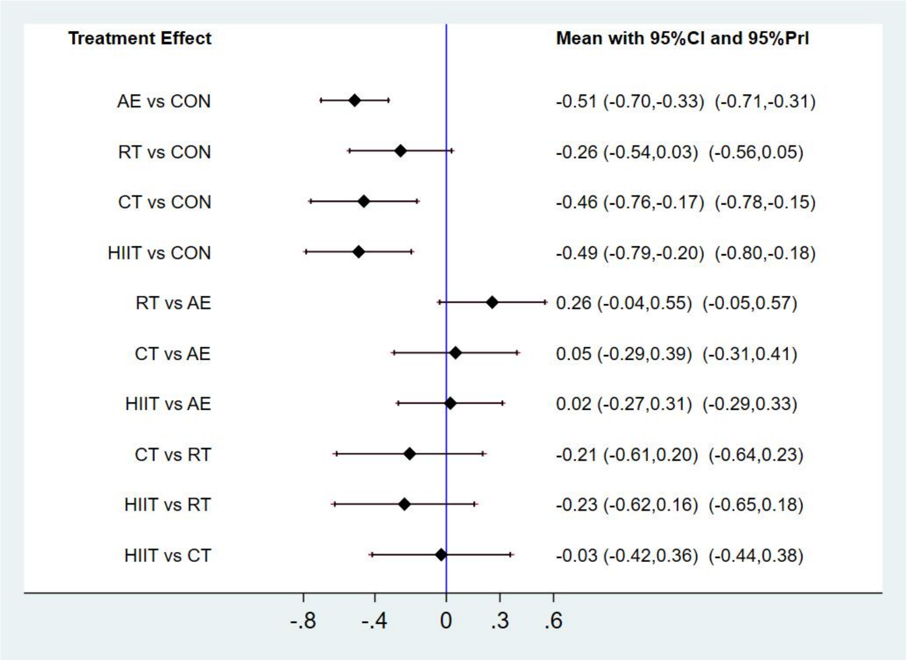

Figure S2. Interval plot of network meta-analysis for body weight. AE, aerobic exercise; RT, resistance training; CT, aerobic combined resistance training; HIIT, high-intensity interval training, CON, control group.

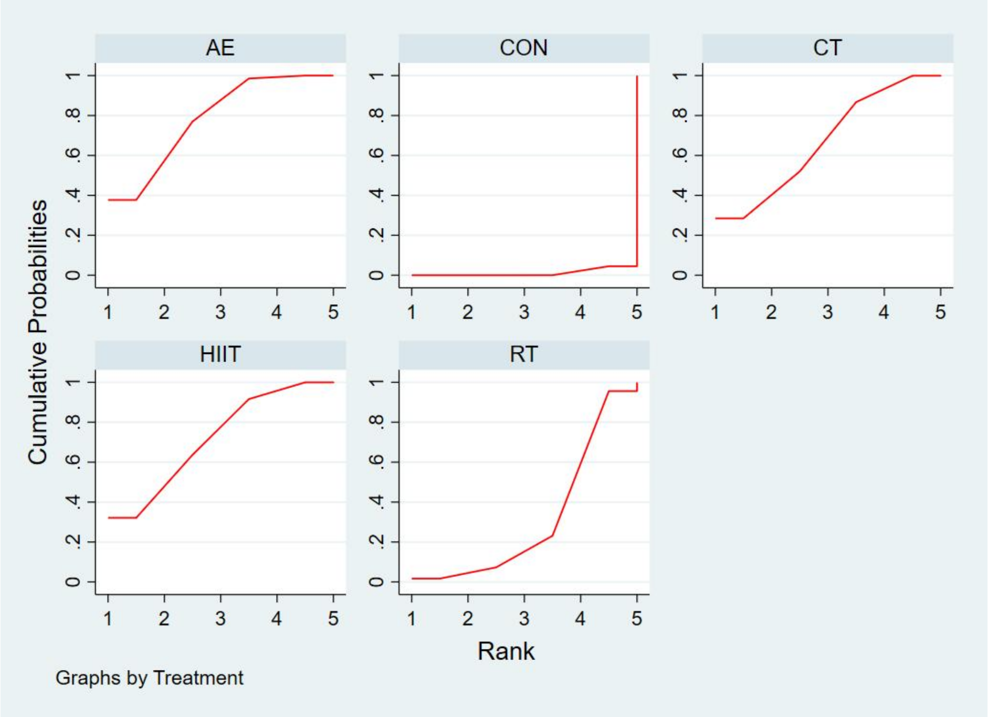

Figure S3. Cumulative ranking probability plots for body weight. AE, aerobic exercise; RT, resistance training; CT, aerobic combined resistance training; HIIT, high-intensity interval training, CON, control group.

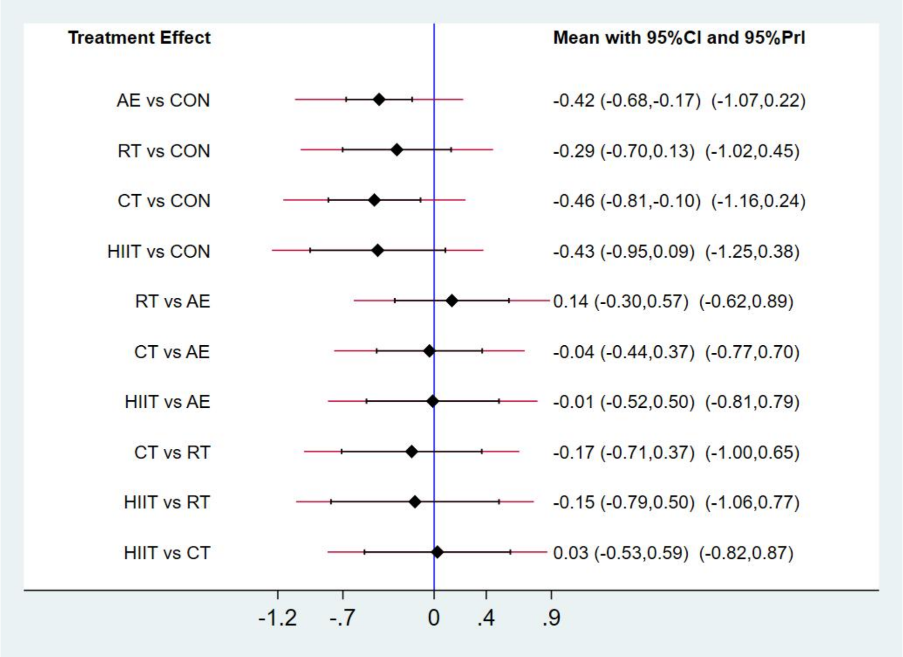

Figure S4. Interval plot of network meta-analysis for body mass index. AE, aerobic exercise; RT, resistance training; CT, aerobic combined resistance training; HIIT, high-intensity interval training, CON, control group.

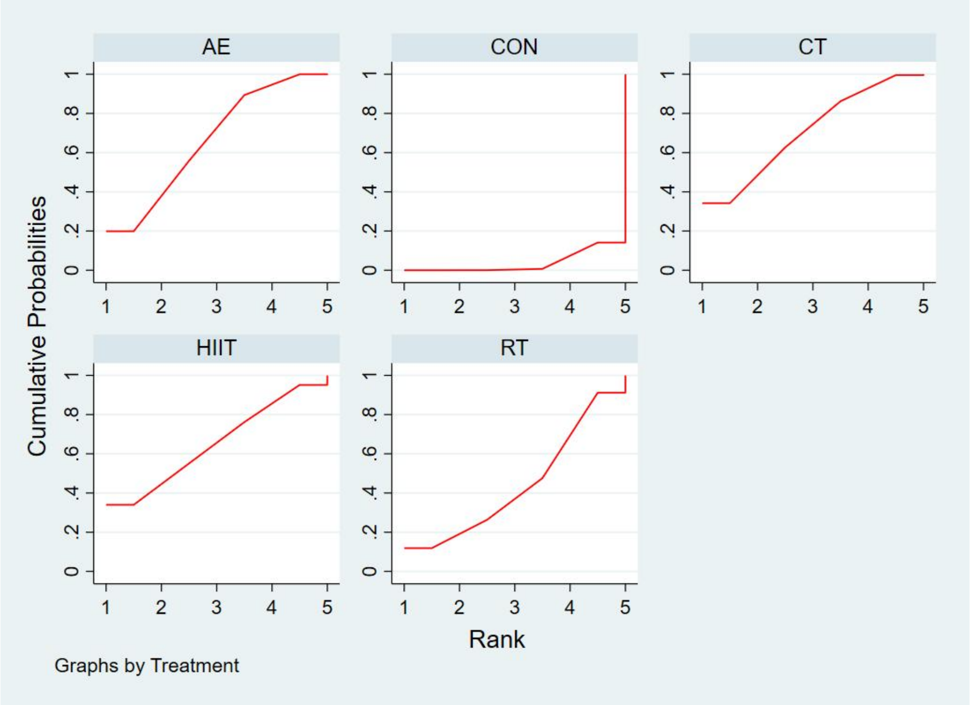

Figure S5. Cumulative ranking probability plots for body mass index. AE, aerobic exercise; RT, resistance training; CT, aerobic combined resistance training; HIIT, high-intensity interval training, CON, control group.

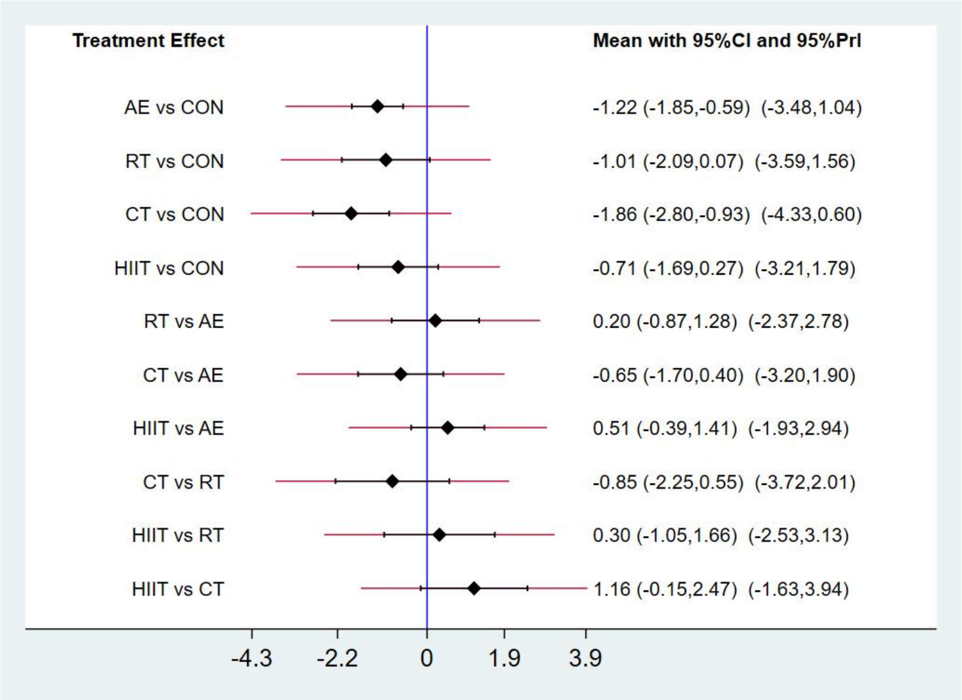

Figure S6. Interval plot of network meta-analysis for waist circumference.AE, aerobic exercise; RT, resistance training; CT, aerobic combined resistance training; HIIT, high-intensity interval training, CON, control group.

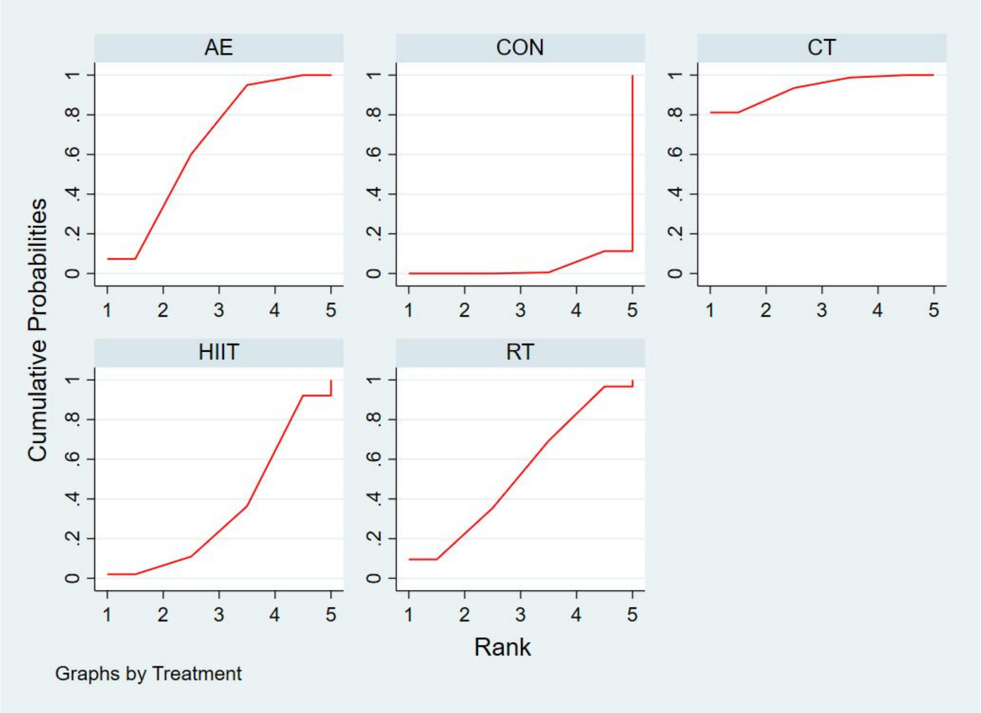

Figure S7. Cumulative ranking probability plots for waist circumference. AE, aerobic exercise; RT, resistance training; CT, aerobic combined resistance training; HIIT, high-intensity interval training, CON, control group.

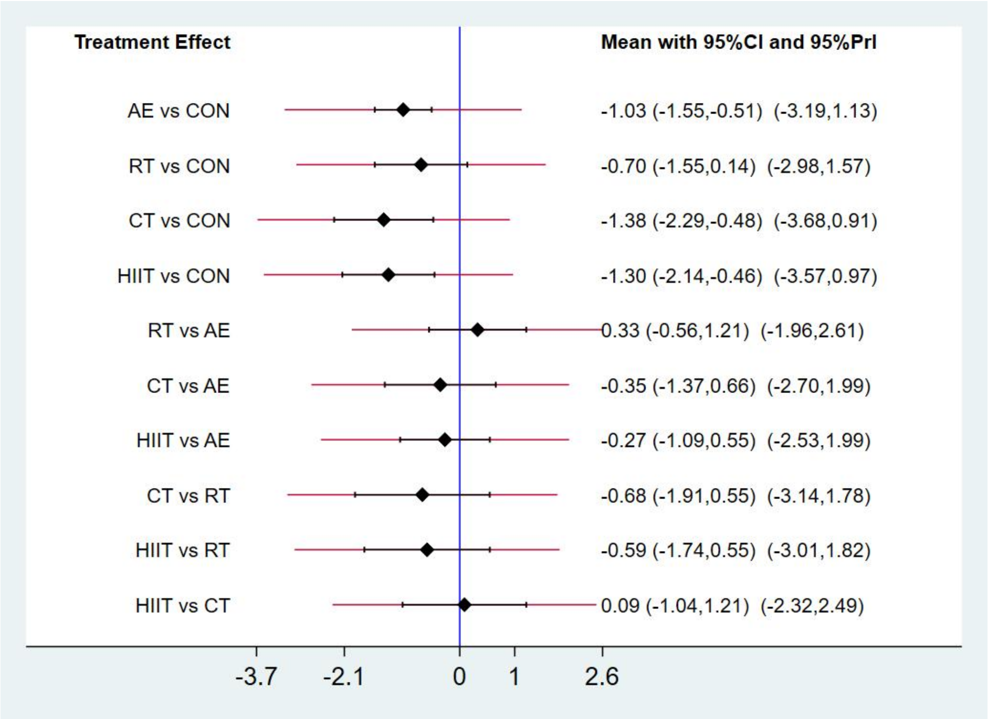

Figure S8. Interval plot of network meta-analysis for percentage body fat. AE, aerobic exercise; RT, resistance training; CT, aerobic combined resistance training; HIIT, high-intensity interval training, CON, control group.

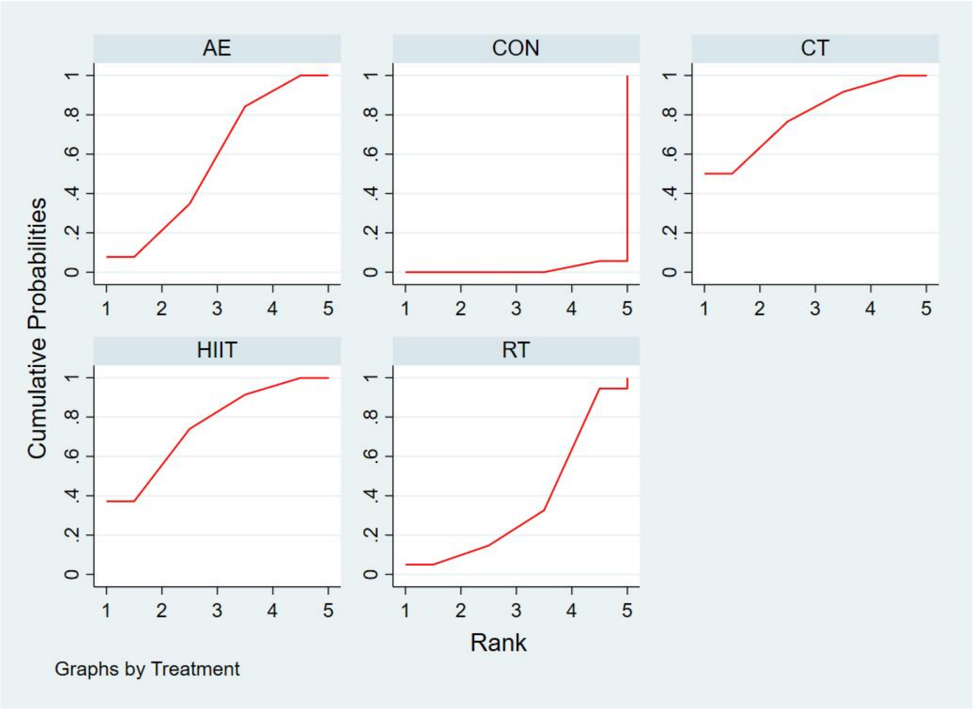

Figure S9. Cumulative ranking probability plots for percentage body fat. AE, aerobic exercise; RT, resistance training; CT, aerobic combined resistance training; HIIT, high-intensity interval training, CON, control group.

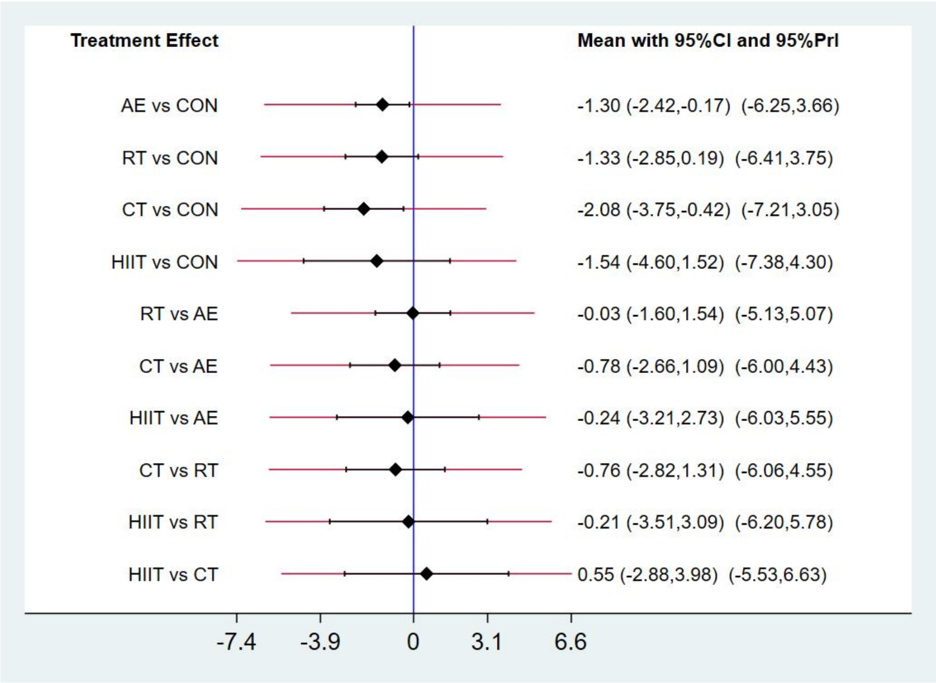

Figure S10. Interval plot of network meta-analysis for tumor necrosis factor-α. AE, aerobic exercise; RT, resistance training; CT, aerobic combined resistance training; HIIT, high-intensity interval training, CON, control group.

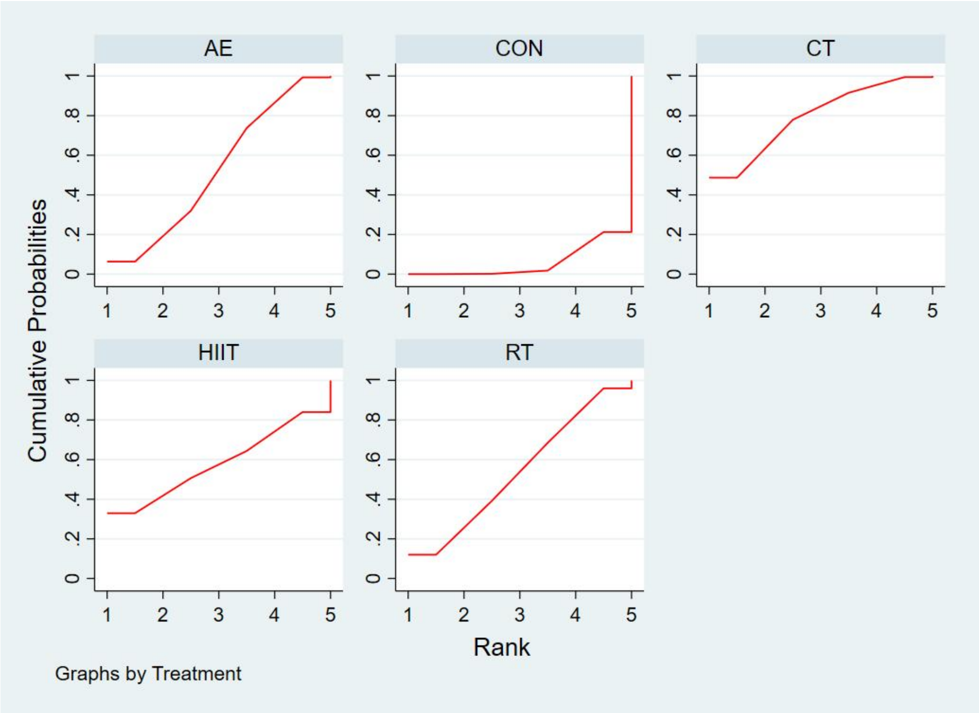

Figure S11. Cumulative ranking probability plots for tumor necrosis factor-α. AE, aerobic exercise; RT, resistance training; CT, aerobic combined resistance training; HIIT, high-intensity interval training, CON, control group.

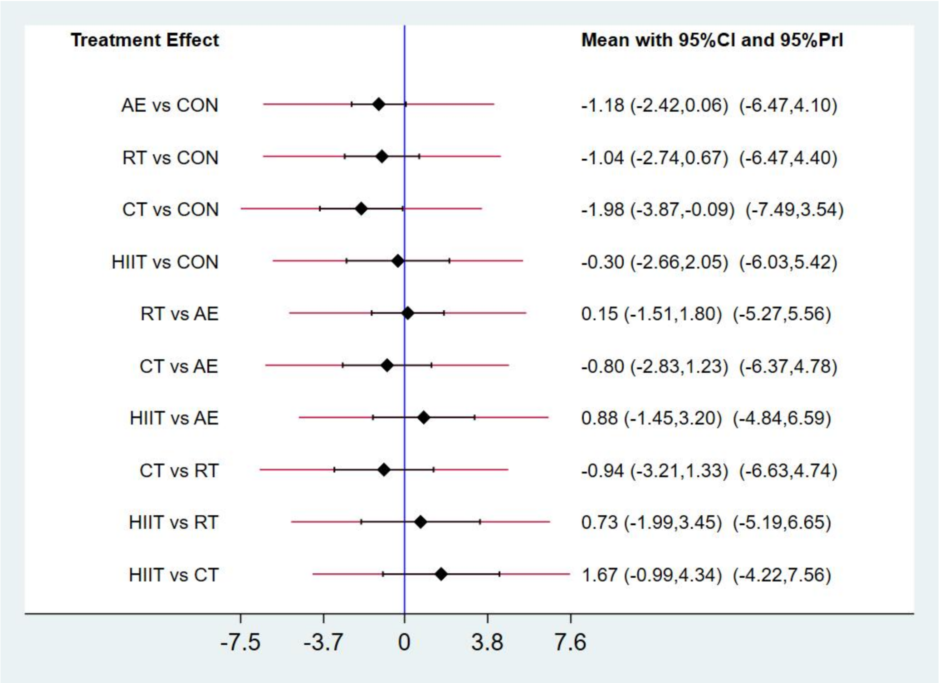

Figure S12. Interval plot of network meta-analysis for interleukin-6. AE, aerobic exercise; RT, resistance training; CT, aerobic combined resistance training; HIIT, high-intensity interval training, CON, control group.

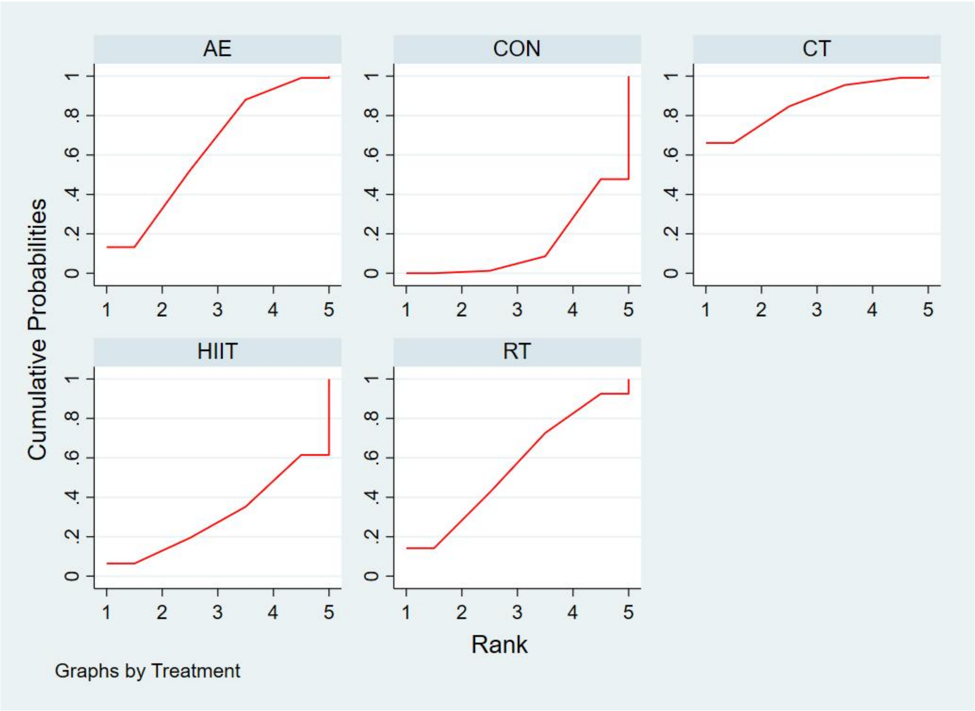

Figure S13. Cumulative ranking probability plots for interleukin-6. AE, aerobic exercise; RT, resistance training; CT, aerobic combined resistance training; HIIT, high-intensity interval training, CON, control group.

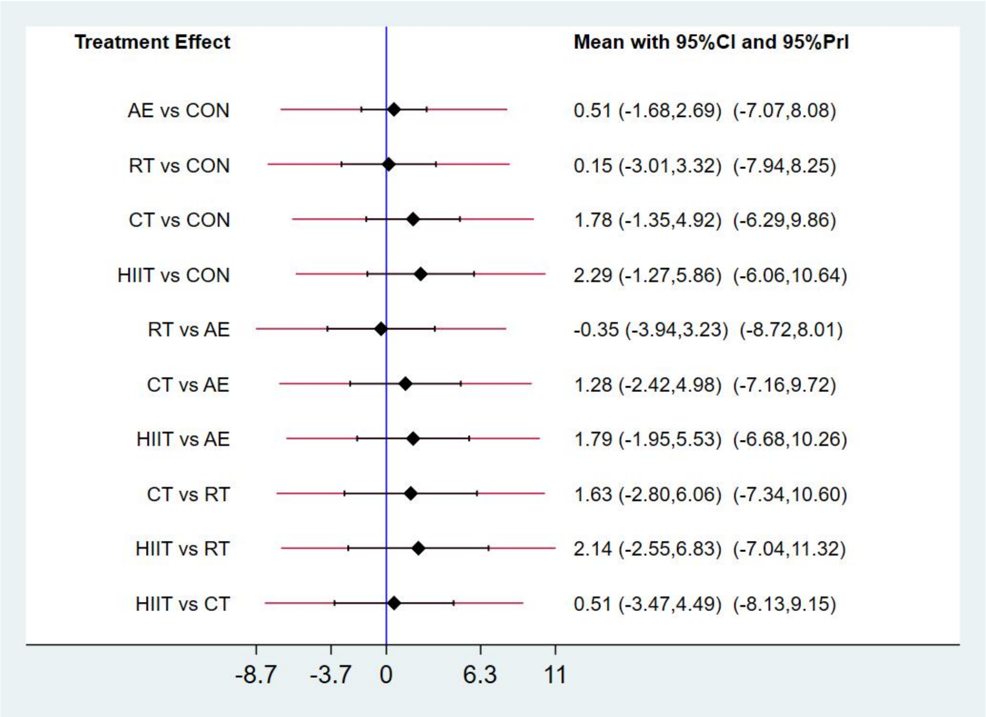

Figure S14. Interval plot of network meta-analysis for adiponectin. AE, aerobic exercise; RT, resistance training; CT, aerobic combined resistance training; HIIT, high-intensity interval training, CON, control group.

Table S6. Ranking of exercise interventions in order of effectiveness.

	BW		BMI		WC		%BF		TNF-α		IL-6	
Exercise	SUCRA	Mean rank	SUCRA	Mean rank	SUCRA	Mean rank	SUCRA	Mean rank	SUCRA	Mean rank	SUCRA	Mean rank
AE	78.3	1.9	66.3	2.3	65.6	2.4	56.7	2.7	52.9	2.9	63.2	2.5
RT	31.9	3.7	44.3	3.2	52.7	2.9	36.7	3.5	53.9	2.8	55.4	2.8
CT	66.8	2.3	70.7	2.2	93.4	1.3	79.6	1.8	79.4	1.8	86.4	1.5
HIIT	71.8	2.1	65.1	2.4	35.4	3.6	75.6	2.0	58	2.7	30.6	3.8
CON	1.1	5.0	3.7	4.9	3.0	4.9	1.4	4.9	5.8	4.8	14.4	4.4

Abbreviations: AE, aerobic exercise; RT, resistance training; CT, aerobic combined resistance training; HIIT, high-intensity interval training; CON, control group; BW, body weight; BMI, body mass index; WC, waist circumference; %BF, percentage body fat; TNF-α, tumor necrosis factor-α; IL-6, interleukin-6.

Note: SUCRA ranges from 0 to 100, where 100 is the most effective therapy and 0 is the worst and ineffective.

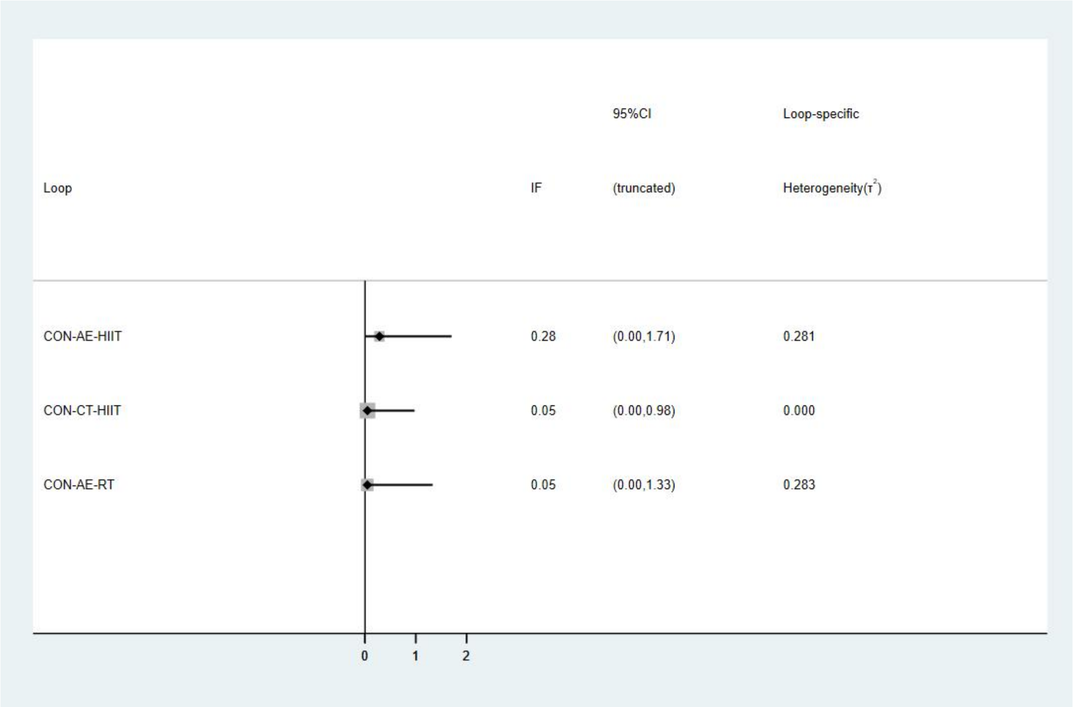

Figure S15. Local inconsistency for body weight.

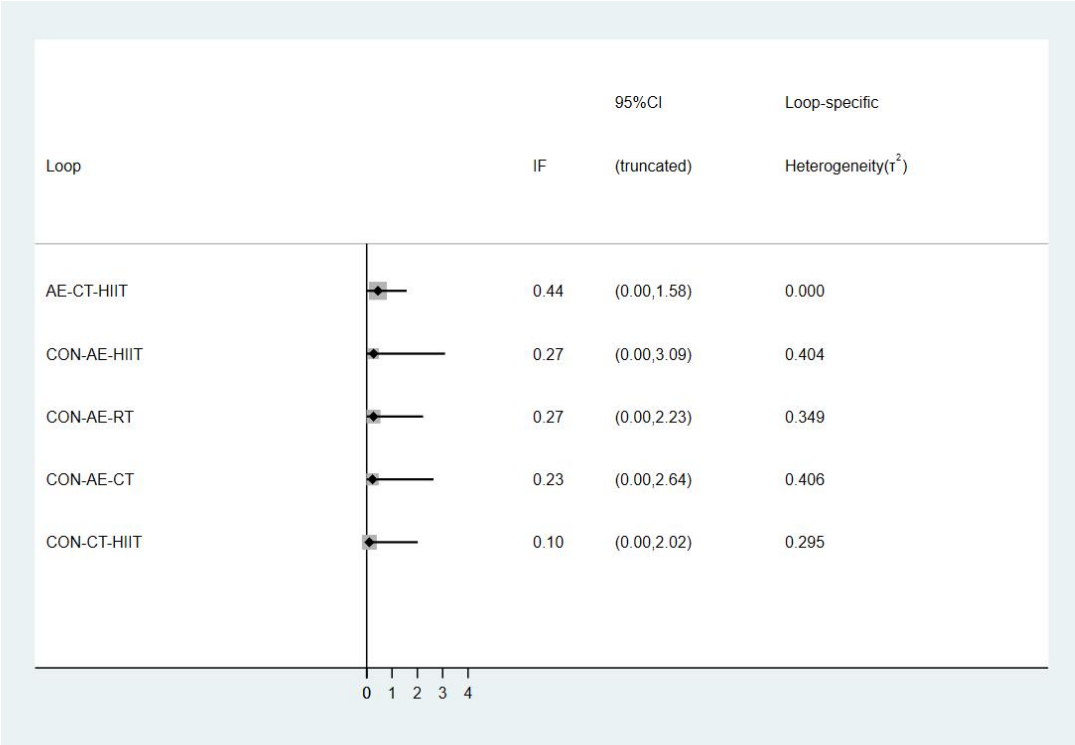

Figure S16. Local inconsistency for body mass index.

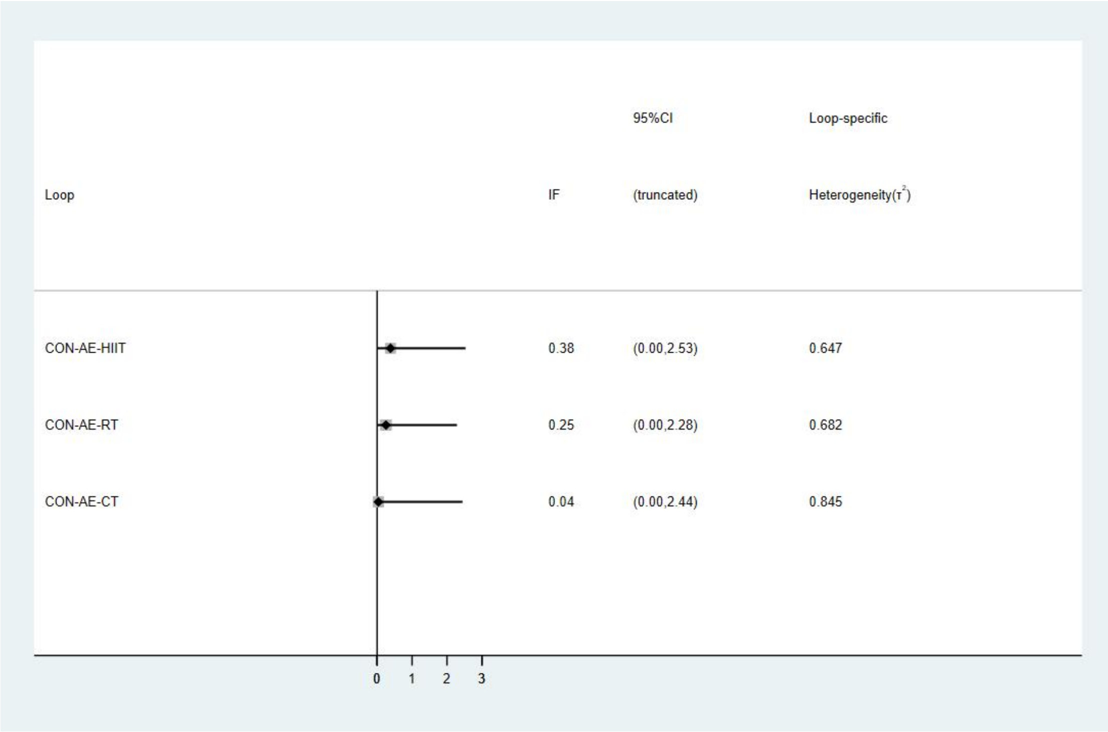

Figure S17. Local inconsistency for waist circumference.

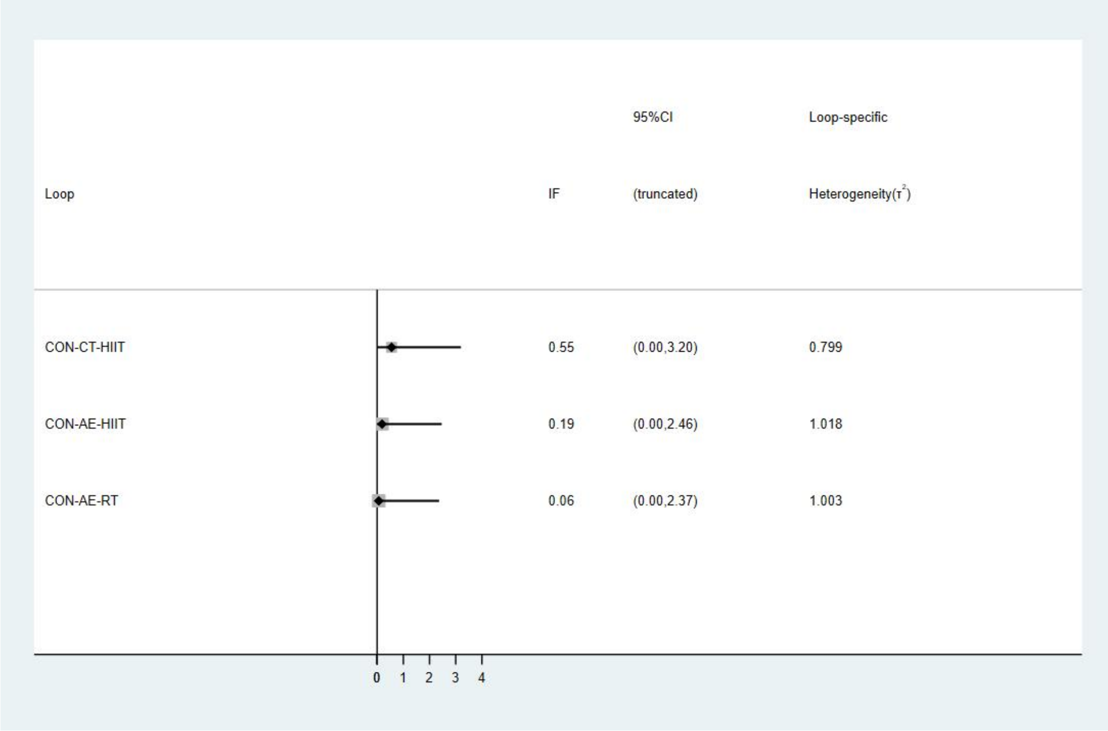

Figure S18. Local inconsistency for percentage body fat.

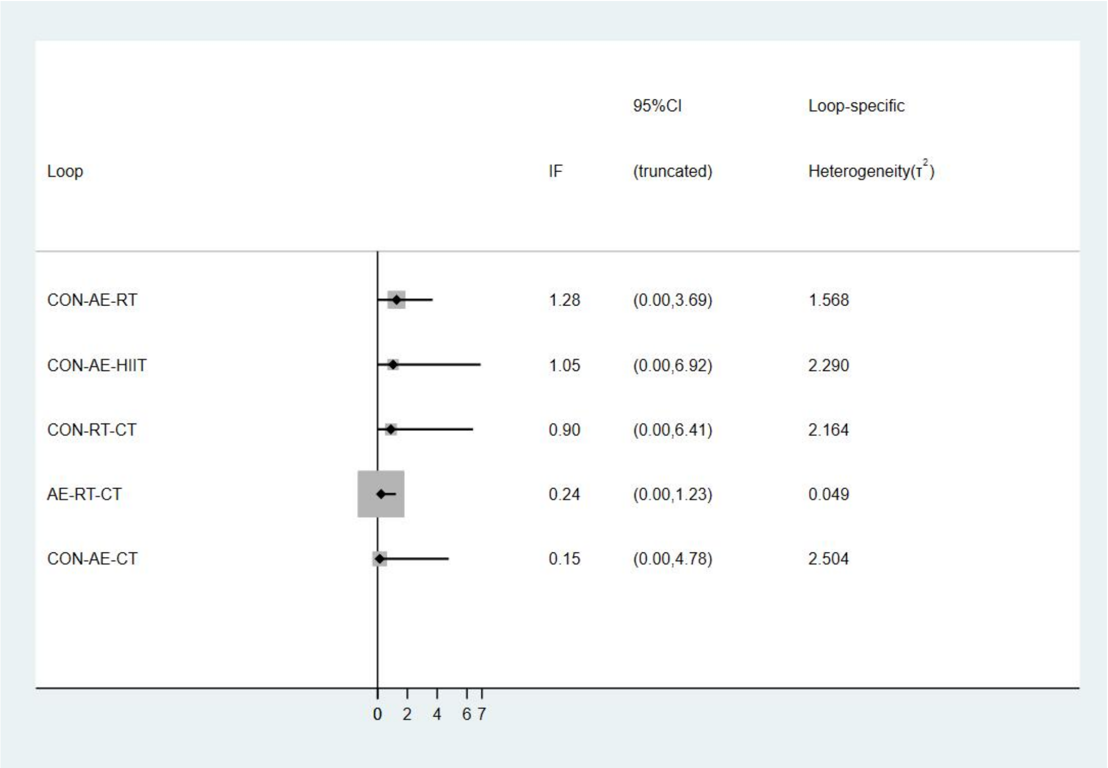

Figure S19. Local inconsistency for tumor necrosis factor-α.

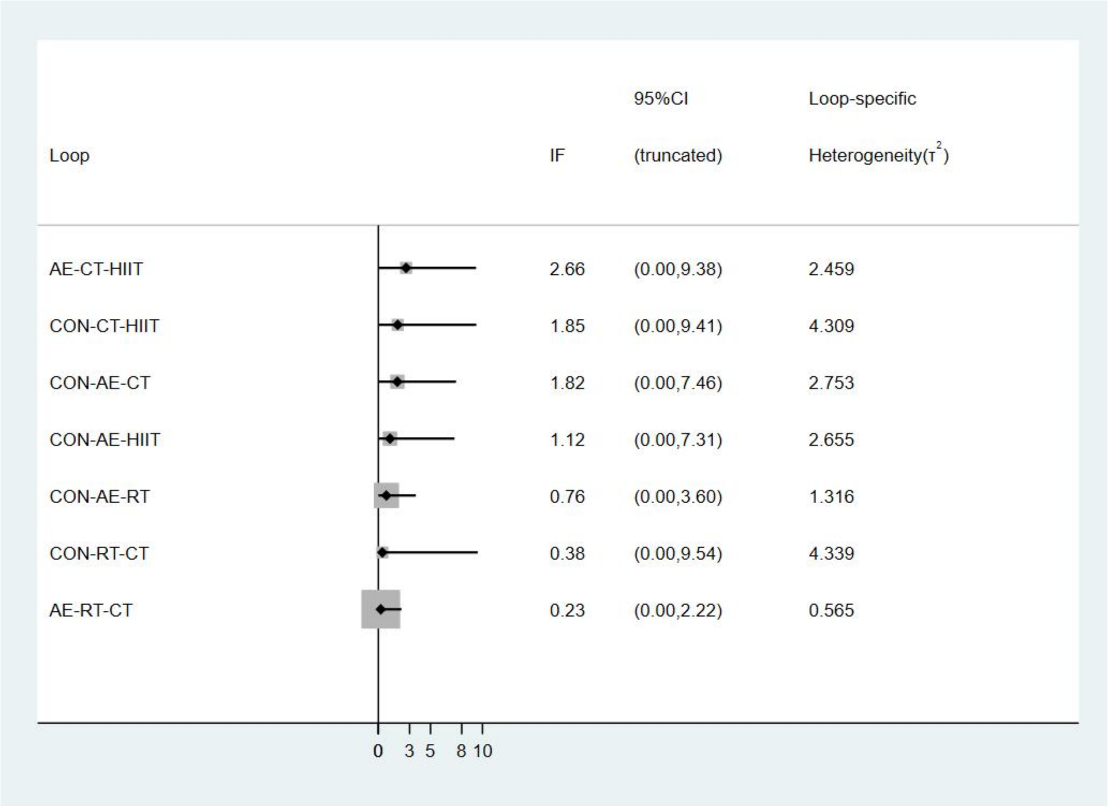

Figure S20. Local inconsistency for tumor necrosis interleukin-6.

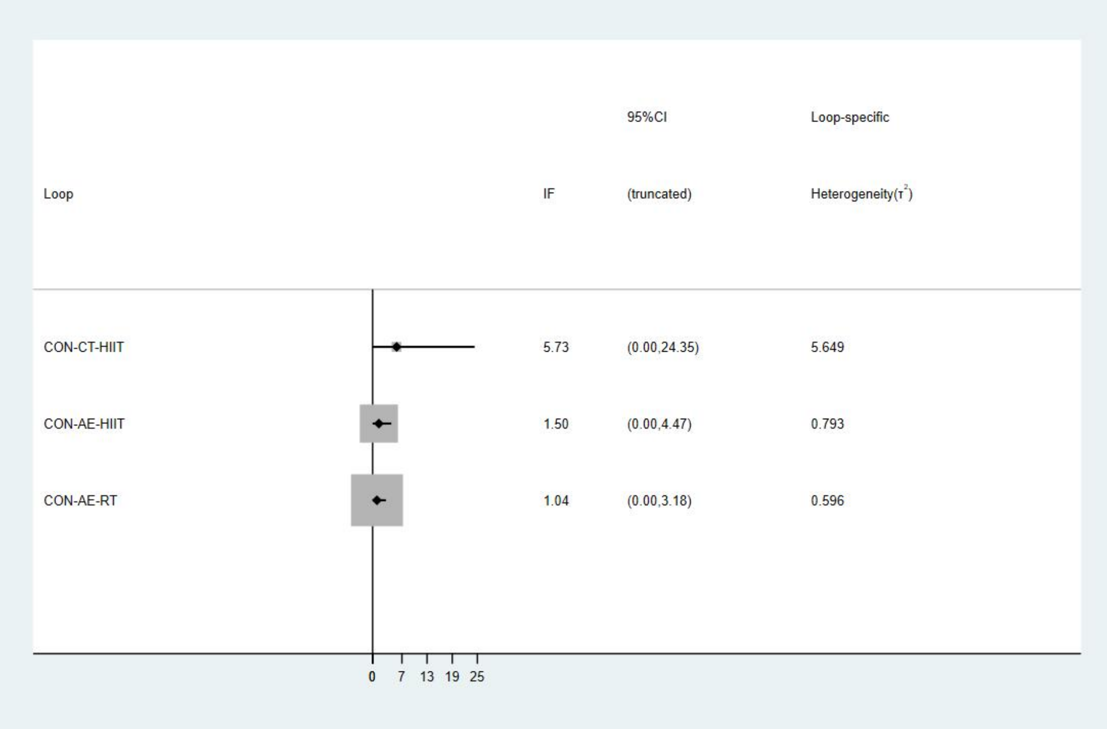

Figure S21. Local inconsistency for tumor necrosis adiponectin.

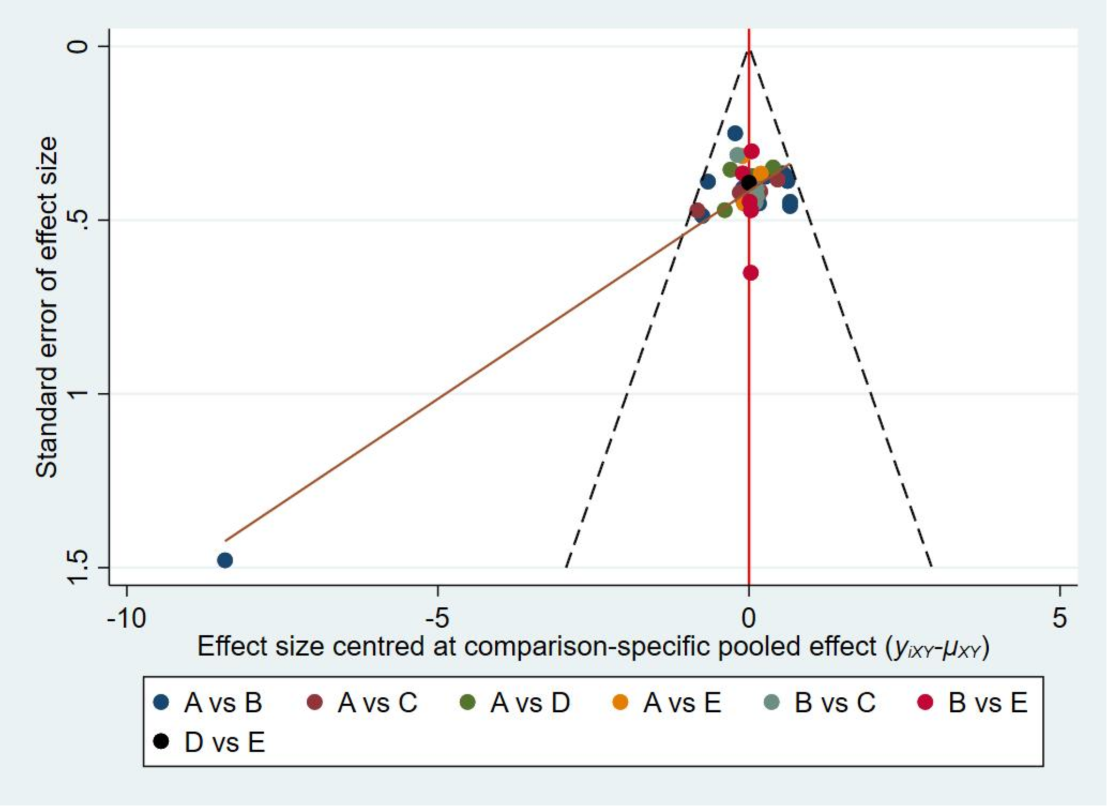

Figure S22. Network meta-analysis funnel plots for body weight.

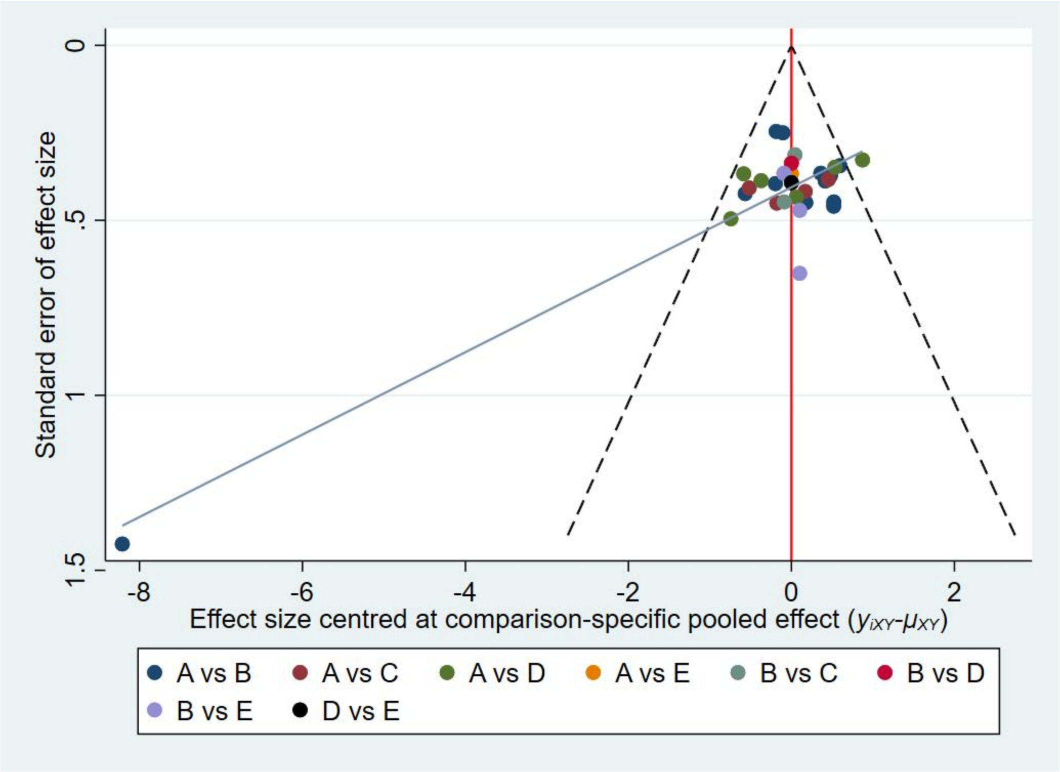

Figure S23. Network meta-analysis funnel plots for body mass index.

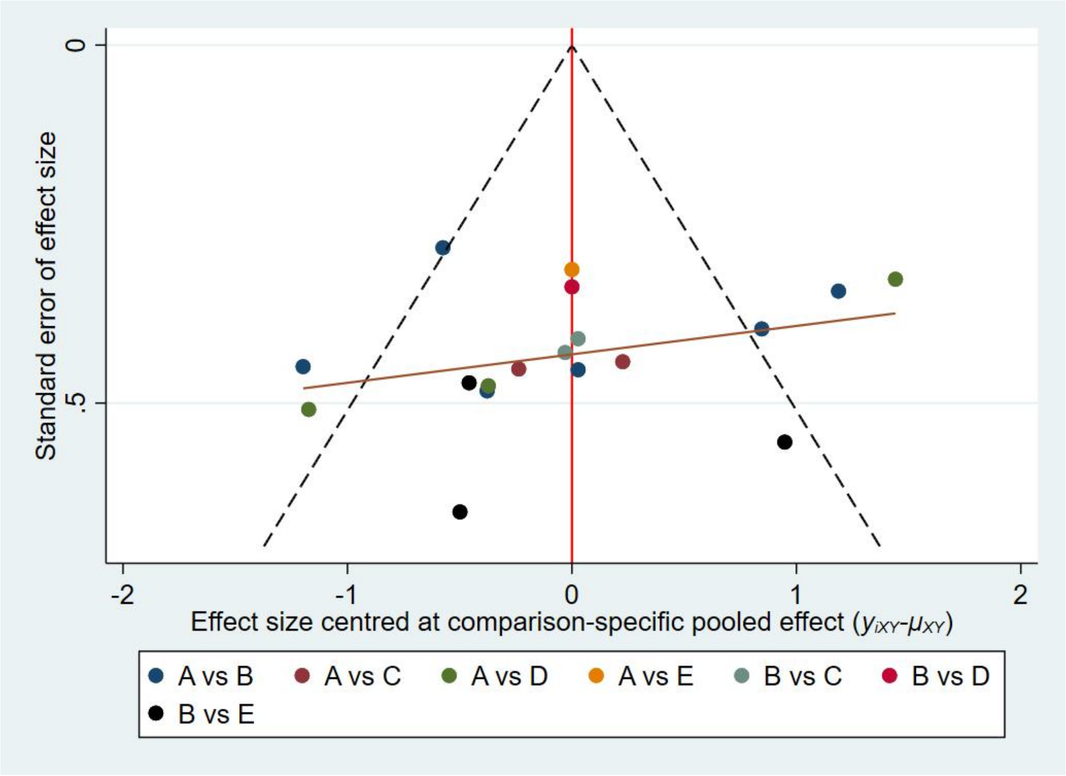

Figure S24. Network meta-analysis funnel plots for waist circumference.

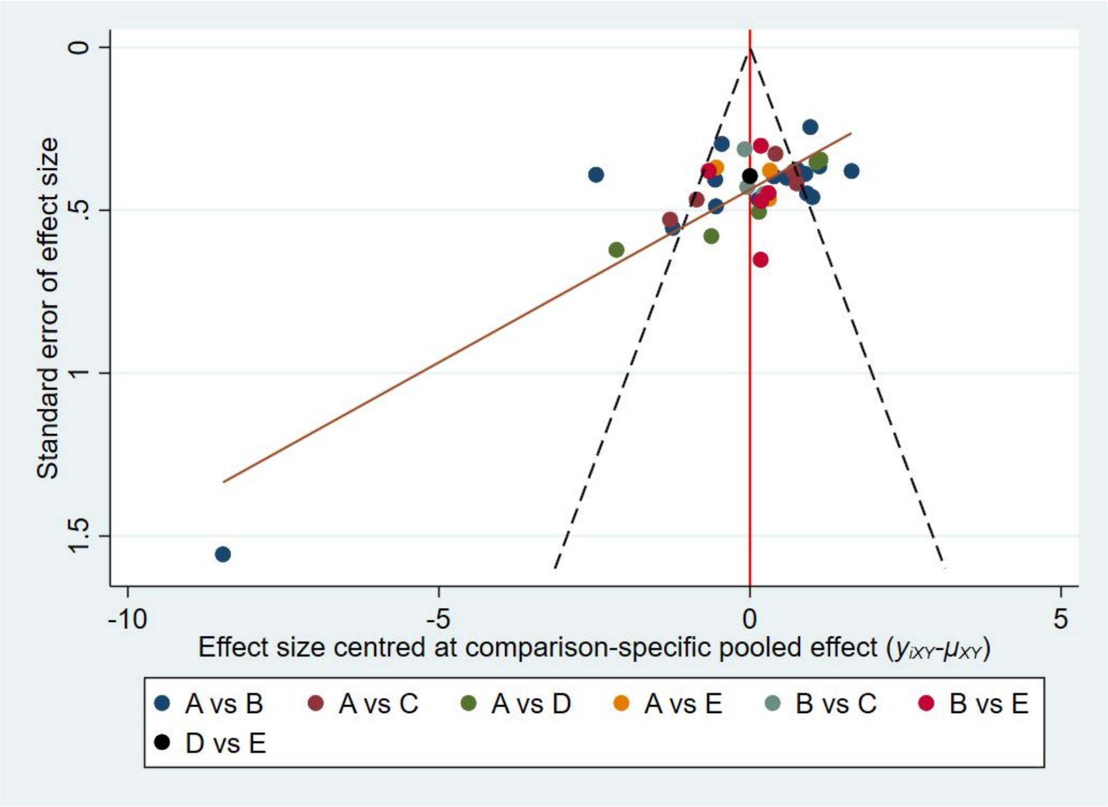

Figure S25. Network meta-analysis funnel plots for percentage body fat.

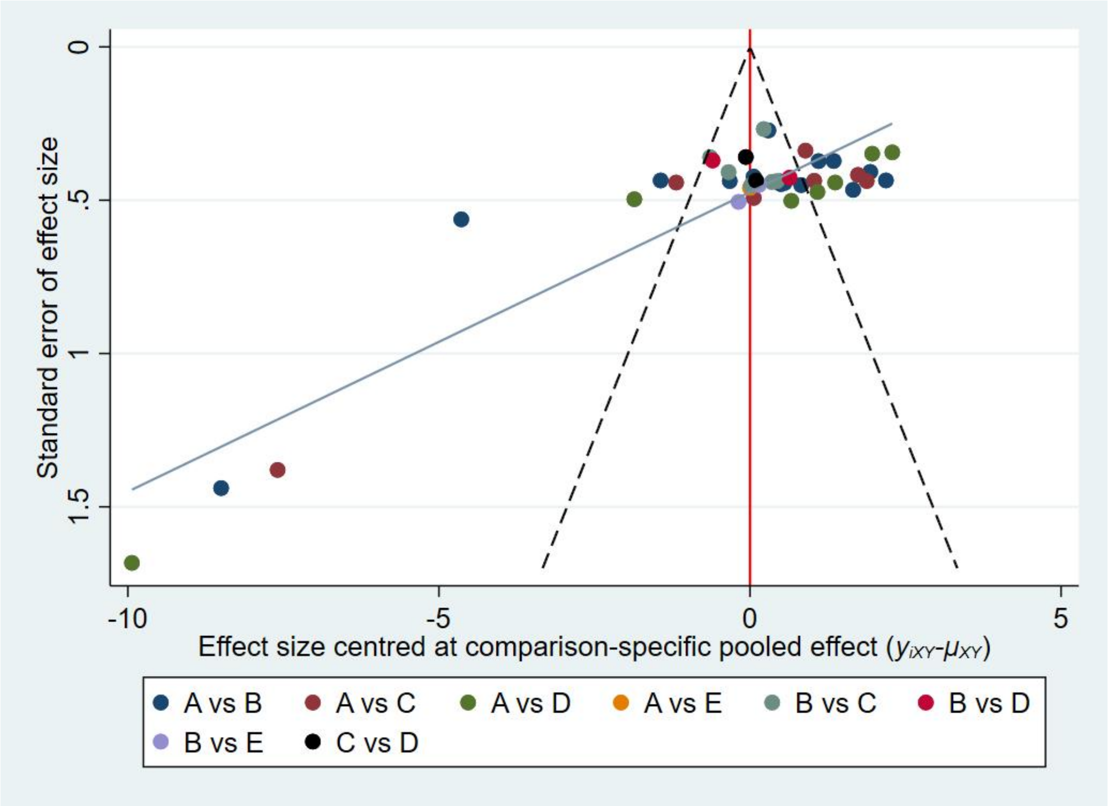

Figure S26. Network meta-analysis funnel plots for tumor necrosis factor-α.

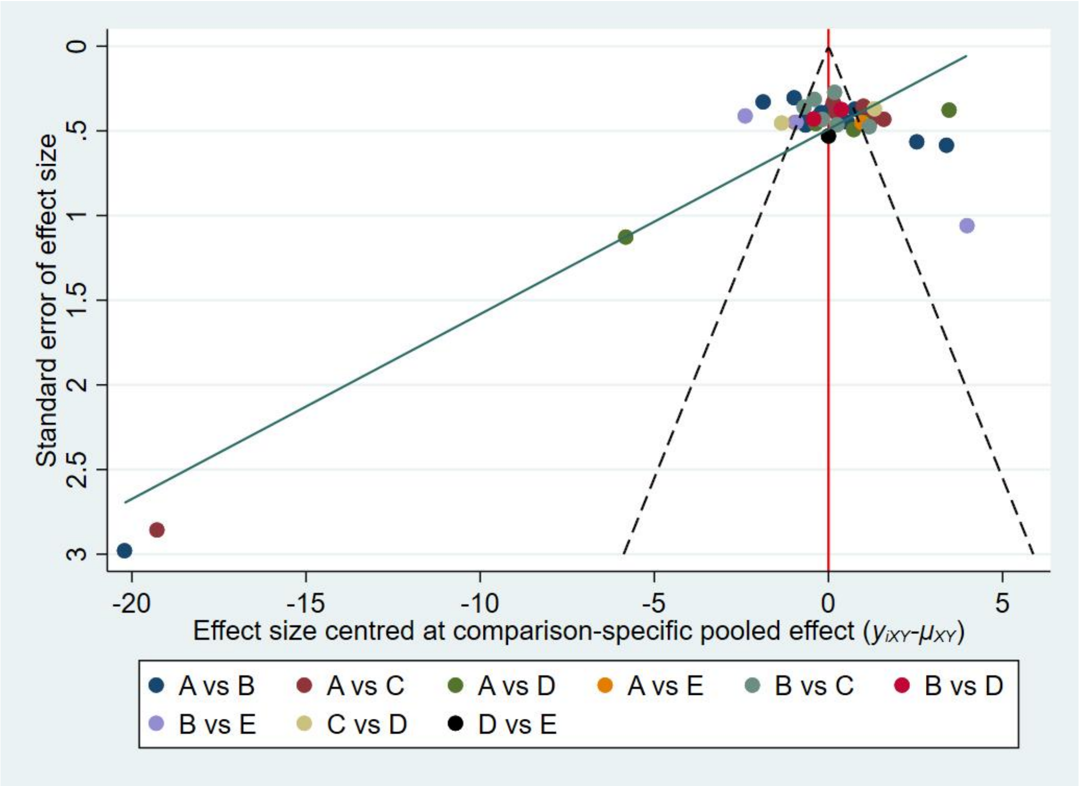

Figure S27. Network meta-analysis funnel plots for interleukin-6.

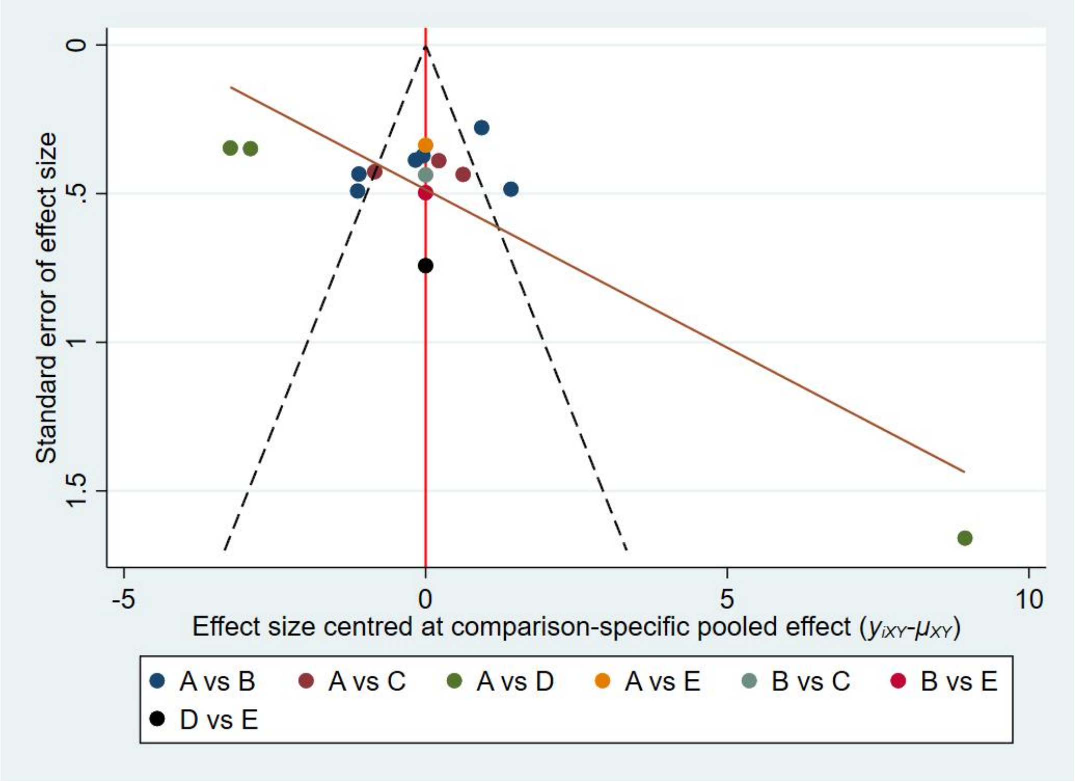

Figure S28. Network meta-analysis funnel plots for adiponectin.

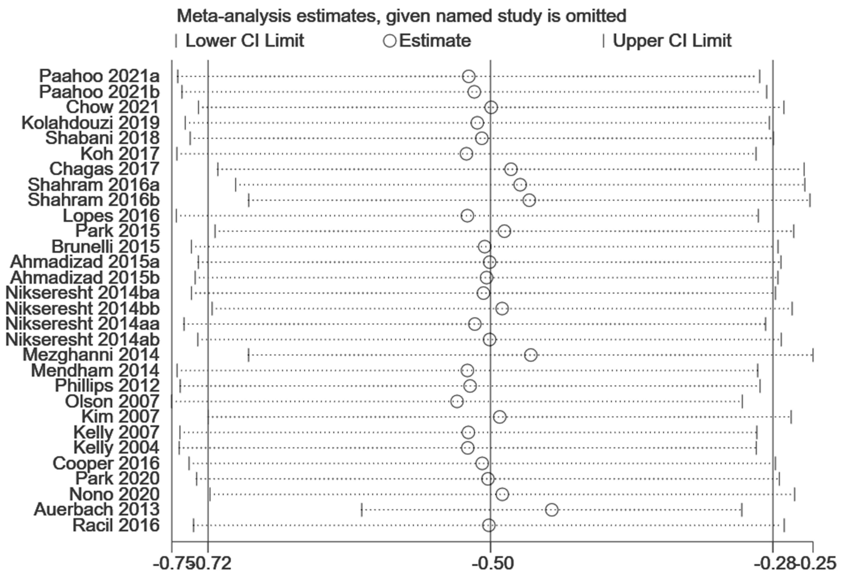

Figure S29. Sensitivity analyses for body weight.

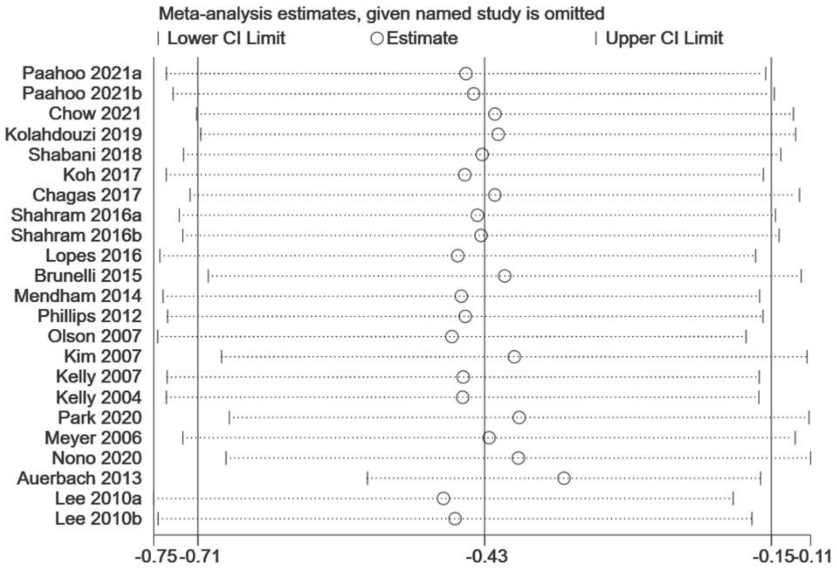

Figure S30. Sensitivity analyses for body mass index.

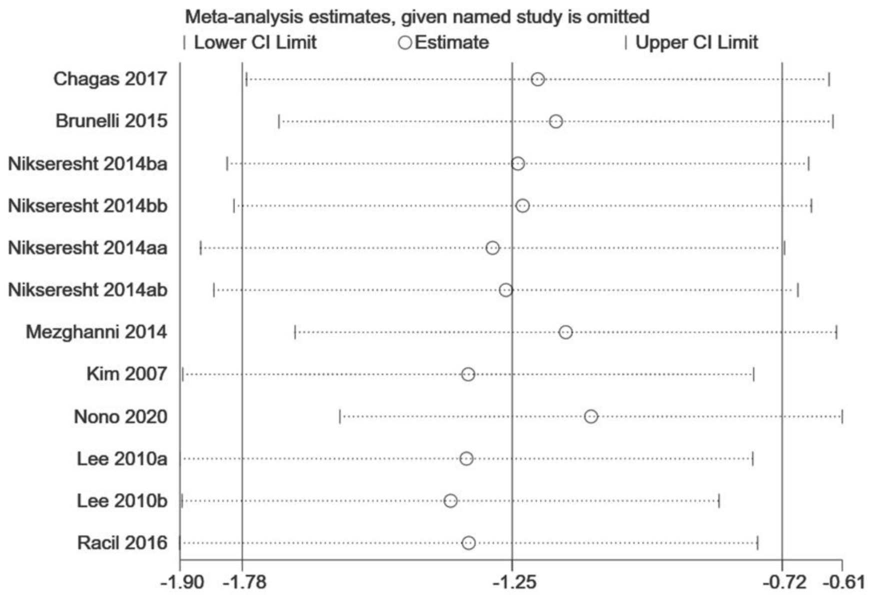

Figure S31. Sensitivity analyses for waist circumference.

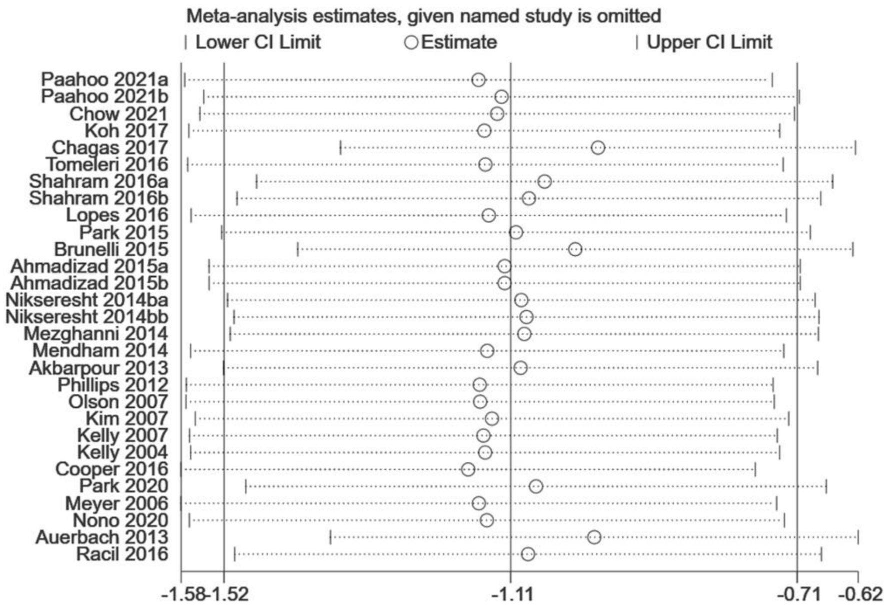

Figure S32. Sensitivity analyses for percentage body fat.

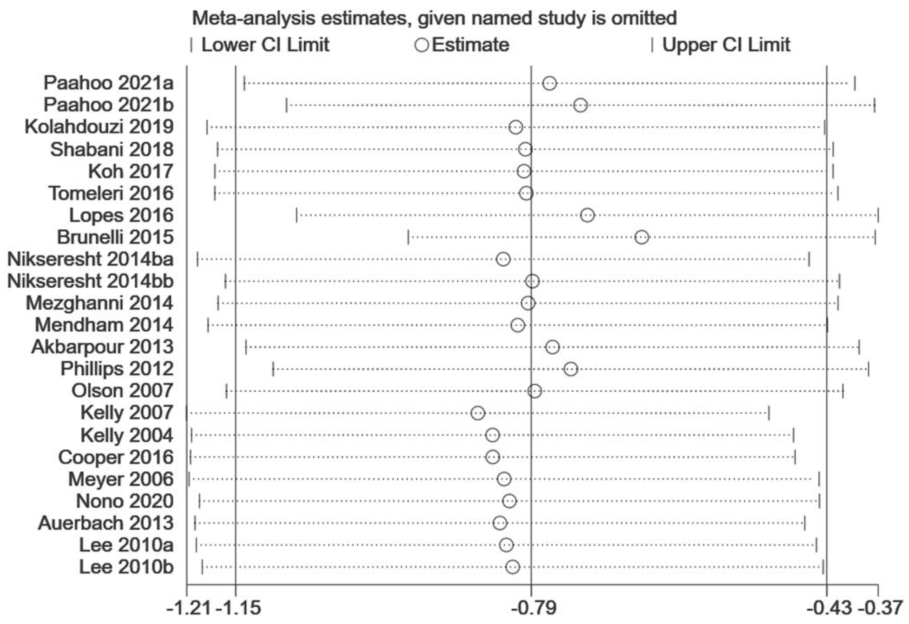

Figure S33. Sensitivity analyses for C-reactive protein.

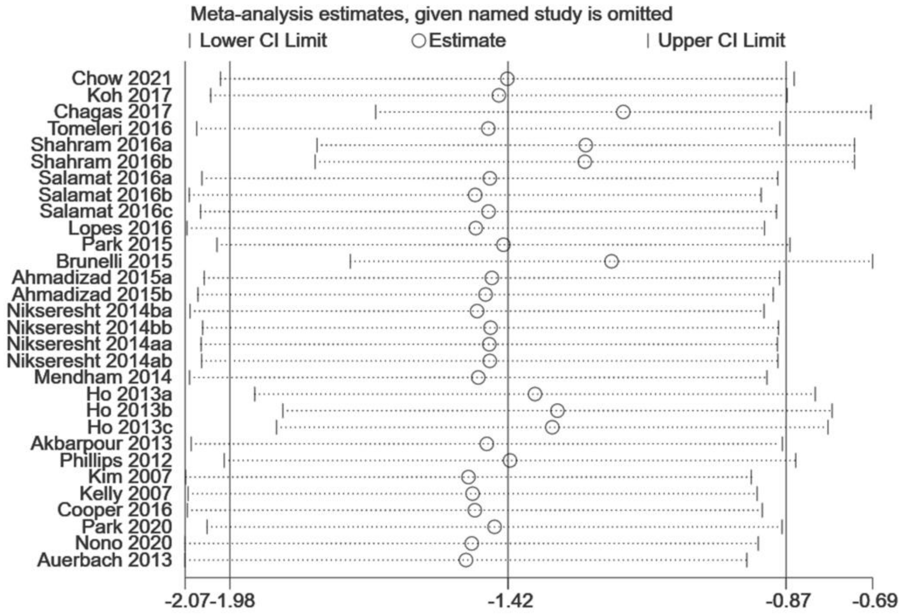

Figure S34. Sensitivity analyses for Tumor necrosis factor-α.

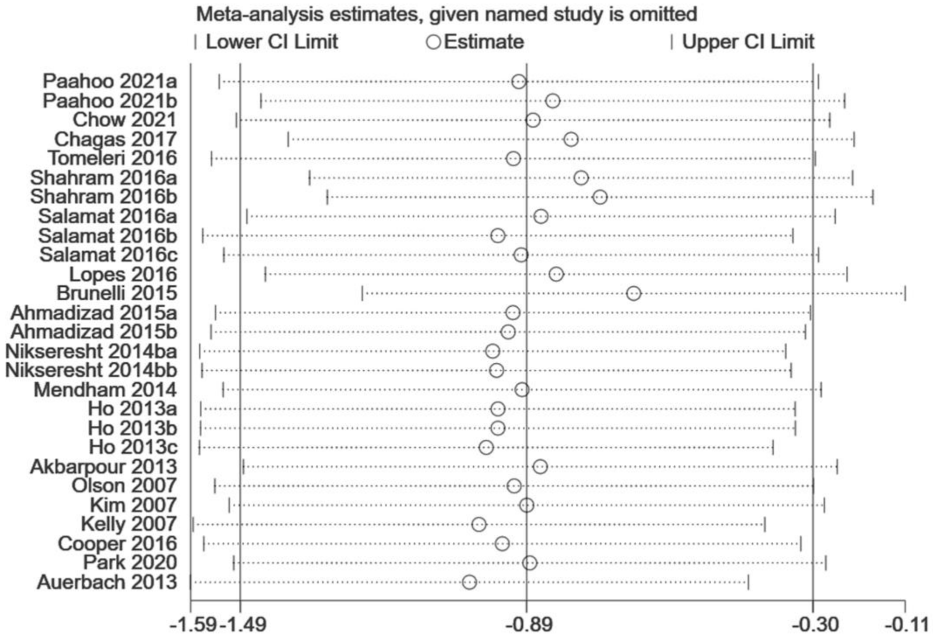

Figure S35. Sensitivity analyses for Interleukin-6.

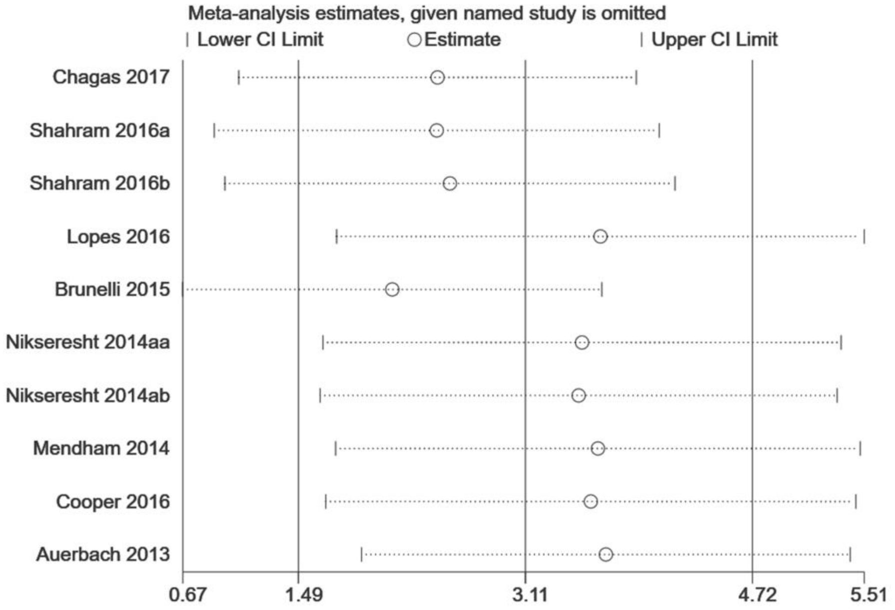

Figure S36. Sensitivity analyses for Interleukin-10.

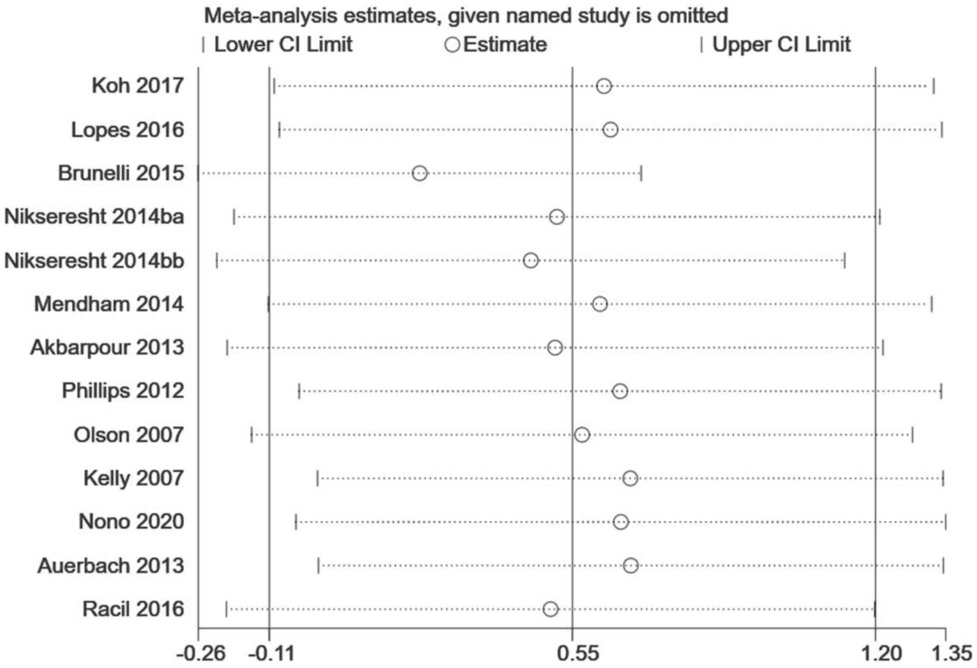

Figure S37. Sensitivity analyses for Adiponectin.
